# Xrn1p acts at multiple steps in the budding-yeast RNAi pathway to enhance the efficiency of silencing

**DOI:** 10.1093/nar/gkaa468

**Published:** 2020-06-05

**Authors:** Matthew A Getz, David E Weinberg, Ines A Drinnenberg, Gerald R Fink, David P Bartel

**Affiliations:** Whitehead Institute for Biomedical Research, Cambridge, MA 02142, USA; Department of Biology, Massachusetts Institute of Technology, Cambridge, MA 02139, USA; Howard Hughes Medical Institute, Cambridge, MA 02142, USA; Whitehead Institute for Biomedical Research, Cambridge, MA 02142, USA; Department of Biology, Massachusetts Institute of Technology, Cambridge, MA 02139, USA; Howard Hughes Medical Institute, Cambridge, MA 02142, USA; Whitehead Institute for Biomedical Research, Cambridge, MA 02142, USA; Howard Hughes Medical Institute, Cambridge, MA 02142, USA; Whitehead Institute for Biomedical Research, Cambridge, MA 02142, USA; Department of Biology, Massachusetts Institute of Technology, Cambridge, MA 02139, USA; Whitehead Institute for Biomedical Research, Cambridge, MA 02142, USA; Department of Biology, Massachusetts Institute of Technology, Cambridge, MA 02139, USA; Howard Hughes Medical Institute, Cambridge, MA 02142, USA

## Abstract

RNA interference (RNAi) is a gene-silencing pathway that can play roles in viral defense, transposon silencing, heterochromatin formation and post-transcriptional gene silencing. Although absent from *Saccharomyces cerevisiae*, RNAi is present in other budding-yeast species, including *Naumovozyma castellii*, which have an unusual Dicer and a conventional Argonaute that are both required for gene silencing. To identify other factors that act in the budding-yeast pathway, we performed an unbiased genetic selection. This selection identified Xrn1p, the cytoplasmic 5′-to-3′ exoribonuclease, as a cofactor of RNAi in budding yeast. Deletion of *XRN1* impaired gene silencing in *N. castellii*, and this impaired silencing was attributable to multiple functions of Xrn1p, including affecting the composition of siRNA species in the cell, influencing the efficiency of siRNA loading into Argonaute, degradation of cleaved passenger strand and degradation of sliced target RNA.

## INTRODUCTION

RNA interference (RNAi), a gene-silencing pathway triggered by double-stranded RNAs (dsRNAs), can play important roles in viral defense, transposon silencing and host gene regulation (1,2). In this pathway, dsRNAs originating from viruses, transposons, or cellular loci are initially processed by the ribonuclease (RNase) III enzyme Dicer into ∼21–23-nucleotide (nt) small interfering RNAs (siRNAs) that are paired to each other with 2-nt 3′ overhangs characteristic of RNase III-mediated cleavage. The siRNA duplexes are then loaded into the RNAi effector endonuclease Argonaute, after which one siRNA strand, designated the passenger strand, is cleaved by Argonaute and discarded (or is occasionally discarded without cleavage), thereby generating the mature RNA-induced silencing complex (RISC). Within RISC, the remaining strand, designated the guide strand, pairs with single-stranded RNAs (ssRNAs) to direct the Argonaute-catalyzed slicing of these target transcripts (1,3–4).

Although conserved in most eukaryotes, RNAi is absent from the model budding-yeast species, *Saccharomyces cerevisiae* (5,6). Indeed, the loss of RNAi has allowed *S. cerevisiae* and some other fungal species the opportunity to harbor Killer, a dsRNA element that encodes a toxin that kills neighboring yeast cells that lack Killer (7). RNAi is present in related budding-yeast species, including *Naumovozyma castellii* and *Vanderwaltozyma polyspora* (formerly *Saccharomyces castelllii* and *Kluyveromyces polysporus*, respectively), which consequently do not have Killer dsRNA (7,8). *Naumovozyma castellii* produces siRNAs that map to hundreds of endogenous loci, a majority of which correspond to transposable elements and Y′ subtelomeric repeats (8). *Naumovozyma castellii* and other budding yeast thought to have retained RNAi possess a non-canonical Dicer gene (*DCR1*) and a canonical Argonaute gene (*AGO1*), which are, respectively, essential for the production and function of siRNAs in *N. castellii* (8). These two genes are also sufficient for the reconstitution of RNAi in *S. cerevisiae* (8).

In species outside the budding-yeast lineage, factors in addition to Dicer and Argonaute support RNAi pathways. For example, RNA-dependent RNA polymerases (RdRPs) are required for RNAi and related pathways in nematodes, some fungi, and plants (9–15). These enzymes synthesize complementary RNAs either to initiate or to amplify the RNAi response (9,16). Other factors are involved in the loading and maturation of RISC. In Drosophila, R2D2 (named for its two dsRNA-binding domains and association with Dicer-2) associates with Dicer-2, and this heterodimer binds to, recognizes the thermodynamic asymmetry of, and facilitates, with the help of Hsc70/Hsp90 chaperone machinery, the loading of siRNA duplexes into Ago2 (17–23). HSP90 has also been implicated in siRNA loading into AGO1 in plants (24). In human cells, TRBP (HIV transactivating response element binding protein), like R2D2 in flies, can help to recruit siRNA-containing Dicer to AGO2 (18,25–26). In the filamentous fungus *Neurospora crassa*, the QIP exonuclease removes the passenger strand during RISC activation (27) and in Drosophila and human, the C3PO endonuclease is reported to have a similar activity that degrades the cleaved passenger strand during RISC maturation (28).

Another factor that can enhance the efficiency of RNAi is the Xrn1 5′-to-3′ exonuclease. Xrn1 is a member of a family of enzymes broadly involved in eukaryotic transcription and RNA metabolism (e.g. processing and degradation) (29). Xrn1 is primarily cytosolic and degrades RNA species possessing a 5′ monophosphate, including mRNA-decay intermediates. Xrn1 orthologs in Arabidopsis and Drosophila enhance RNAi by degrading the 3′ slicing products of RISC (30,31). Likewise, in human cells, XRN1 degrades the 3′ slicing fragments and acts together with the exosome to degrade the 5′ slicing fragments of mRNAs targeted by siRNAs (32). By degrading the slicing products of RISC, Xrn1 orthologs likely relieve product inhibition and facilitate multiple turnover of the enzyme. In *Caenorhabditis elegans*, *xrn-1* has also been implicated as being involved in RNAi (33), and its knockdown is associated with the accumulation of certain passenger strands in the microRNA (miRNA) pathway (34), an RNA-silencing pathway that derives from the more basal RNAi pathway (35).

Although the only known protein components of the budding-yeast RNAi pathway were Dicer and Argonaute (Dcr1p and Ago1p), we reasoned that, as observed in animals, plants and other fungi, one or more additional factors might be important for either siRNA production, duplex loading, RISC maturation, or silencing efficiency in yeast. If such a factor did exist, the ability to reconstitute RNAi in *S. cerevisiae* by adding only *DCR1* and *AGO1* indicated that the additional factor either was not essential for RNAi or had other functions in budding yeast, leading to its retention in *S. cerevisiae* after loss of the RNAi pathway. Based on the success of genetic screens and selections in other systems, including Arabidopsis, *C. elegans* and Drosophila, which provided early insight into the core components of the RNAi pathway and identified additional factors that influence RNAi efficacy (36–46), we implemented a genetic selection in *N. castellii* to identify mutants with reduced RNAi activity. This selection identified mutants in the gene encoding Xrn1p. Employing RNA sequencing and biochemical assays, we found Xrn1p acts at multiple steps of the pathway to enhance the efficiency of RNAi in budding yeast. These steps included affecting siRNA populations and loading of siRNA duplexes into Argonaute—steps for which it had not been previously reported to play a role in any species.

## MATERIALS AND METHODS

### Growth conditions and genetic manipulations

*Naumovozyma castellii* was grown at 30°C on standard *S. cerevisiae* liquid and solid media (YPD and SC), unless otherwise indicated. Transformations were performed as described (8), with the following modifications: The transformation mix (cells + DNA + PEG + lithium acetate) was incubated at 23°C for 30 min with rotation and without dimethyl sulfoxide. The mixture was then incubated at 37°C for 20 min, resuspended and plated on selective media.

### Plasmid and strain construction

Plasmid and strain construction is detailed in the supplementary materials, and lists of plasmids and strains generated in this study are provided (Supplementary Tables S5 and S6).

### EMS mutagenesis and selection

Mutagenesis of DPB537, the *N. castellii* genetic-selection strain, with ethyl methane sulfate (EMS) was carried out as described (47), except scaled-up 10-fold. An overnight YPD culture of *N. castellii* was used to inoculate a large YPD (∼150 ml) culture that was grown until OD_600_ 1.0, split equally into aliquots that contained ∼2.1 × 10^9^ cells, one of which was incubated with 300 μl EMS and the other which was incubated with 300 μl sodium phosphate buffer. The OD_600_ of each culture was measured and was used to calculate the dilutions needed to plate 100 mutagenized or control cells on separate YPD plates to determine the death rate of cells in the mutagenesis. Mutagenized cells were split into four equal aliquots and plated on SGal – His, – Leu, – Ura (drop-out media); SD – His, – Leu, – Ura (drop-out media); SM (synthetic minimal) + 2% galactose + Lys (SGal + Lys) (drop-in media); and SM (synthetic minimal) + 2% glucose + Lys (SD + Lys) (drop-in media) agar plates (245 × 245 mm). These plates were incubated at 25°C until colonies formed.

### Flow cytometry and mating of RNAi mutant strains

A culture of each mutant strain that had grown on selective media was inoculated in 200 μl inducing SC media (2% galactose) in a 96-well plate and grown to saturation. Fresh cultures were then inoculated at OD_600_ 0.2 with cells from the saturated cultures and were grown until all strains were in log phase (∼6 h). Cells were analyzed using FACSCalibur (BD Biosciences), and data were processed with CellQuest Pro (BD Biosciences) and FlowJo (Tree Star). For each experiment, a gate for eGFP fluorescence was defined using the wild-type (WT) genetic-selection strain (DPB537) as a negative control to define the boundaries of the gate. Strains for which > 0.5% of the population was in the eGFP-positive gate were carried forward for complementation analysis.

**Figure 1.**
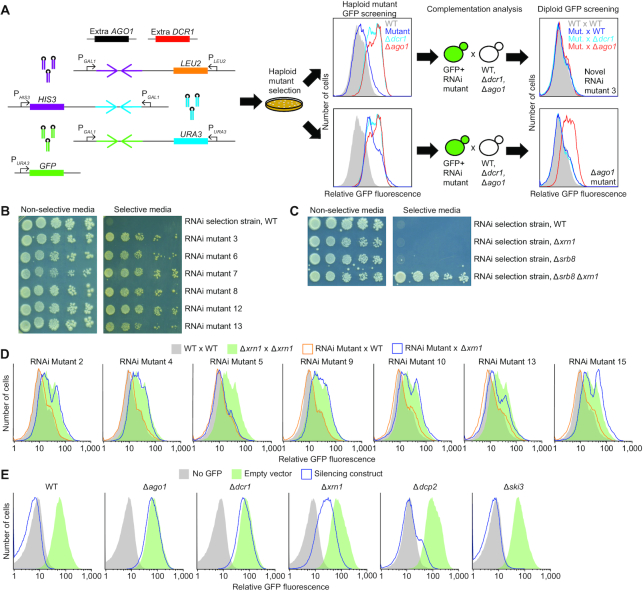
Genetic selection identifying *XRN1* as an enhancer of the RNAi pathway in budding yeast. (**A**) Schematic of the genetic selection. At the left are the six genomic insertions of the parental selection strain. Two of these insertions provided an additional copy of the *AGO1* and the *DCR1* genes (black and red boxes, respectively), each added under control of their native promoters. Three of the insertions provided silencing constructs against *HIS3*, *URA3* and *GFP* genes (purple, blue and green convergent arrows, which indicate regions giving rise to palindromic transcripts that form hairpins) under control of the *Saccharomyces cerevisiae GAL1* promoter (P*_GAL1_*). These three insertions also provided *S. cerevisiae LEU2*, *HIS3* and *URA3* genes (orange, purple and blue boxes, respectively) under control of their *S. cerevisiae* promoters, which replaced their endogenous *Naumovozyma castellii* orthologs. The sixth insertion provided an exogenous *GFP* gene (green box) under control of the *S. cerevisiae URA3* promoter. The haploid selection strain was mutated with EMS, and mutants able to grow on media that lacked Leu, His and Ura were isolated and screened by flow cytometry for their ability to silence *GFP*. Mutant strains able to silence *GFP* were subjected to a complementation analysis in which they were mated with *N. castellii* WT, Δ*ago1* and Δ*dcr1* strains, and the resulting diploid strains were screened by flow cytometry for their ability to silence *GFP*. Strains for which the ability to silence *GFP* was restored in each of the three matings were carried forward as RNAi mutant strains, with the complementation analysis indicating that they each had one or more recessive mutations that reduced silencing and fell outside of *AGO1* and *DCR1*. (**B**) Growth of RNAi mutant strains but not the parental strain on selective media. Representative RNAi mutant strains were serially diluted, plated on either non-selective media (SC (2% galactose)) or selective media (SGal – His, – Leu, – Ura), and allowed to grow for 12 days. For each strain, 10^5^ cells were plated in the leftmost spot, and dilutions were each 10-fold. (**C**) Ability of mutations in both *XRN1* and *SRB8* to confer growth of the selection strain on selective media. Assays compared the growth of single mutant strains (Δ*xrn1* or Δ*srb8*) to that of the double-mutant strain (Δ*srb8* Δ*xrn1*), which were each constructed in the background of the selection strain. Cells were allowed to grow for 10 days; otherwise, as in B. (**D**) Results of complementation analysis of RNAi mutant strains that had mutations in *XRN1*. Shown are histograms of GFP fluorescence measured by flow cytometry in WT × WT (gray), Δ*xrn1* x Δ*xrn1* (green), RNAi mutant × WT (orange) and RNAi mutant x Δ*xrn1* (blue) diploid strains. All strains were induced. (**E**) An intermediate effect on *GFP* silencing observed upon disruption of *XRN1*. Shown are histograms of GFP fluorescence measured by flow cytometry in WT, Δ*ago1*, Δ*dcr1*, Δ*xrn1*, Δ*dcp2* and Δ*ski3* haploid strains that either lacked the *GFP* gene (gray), contained *GFP* but had no silencing construct (green), or contained both *GFP* and a *GFP*-silencing construct (blue). All strains were induced.

Matings of candidate *N. castellii* RNAi-mutant strains with WT (DPB079), Δ*ago1* (DPB325), Δ*dcr1* (DPB534) and Δ*xrn1* (DPB535) strains were performed by mixing equal volumes (100 μl) of saturated YPD cultures in a round-bottom 96-well plate and incubating at 30°C for 24 h. Diploid cells were then selected by plating these mixed saturated cultures on SD – Ade, – Lys agar plates (for RNAi mutants × DPB325) or SD – Met, – Lys agar plates (for all other matings). Flow cytometry was performed as with the haploid mutant strains, except with no gating for eGFP positive cells. Diploid strains presented in Figure 1D were created from matings of the WT genetic-selection strain (DPB537) and its derived RNAi-mutant strains with a WT strain (DPB079) or a Δ*xrn1* strain (DPB535) of the opposite mating type. The diploid *XRN1-*knockout strain was created by mating strain DPB541 with strain DPB510.

### Genome sequencing and analysis

Genomic DNA was isolated as described (48) from saturated YPD cultures of the *N. castellii* genetic-selection strain (DPB537) and RNAi-mutant strains. DNA concentration was calculated using the Qubit dsDNA HS (High Sensitivity) Assay Kit (Invitrogen Q32854) with an Invitrogen Qubit Fluorometer. Genomic DNA libraries were prepared with either the Nextera DNA Library Preparation Kit (Illumina) or the QIAseq FX DNA Library Kit (QIAGEN 180475) and sequenced using Illumina SBS. Single-end sequencing was performed on the HiSeq platform, and paired-end sequencing was performed on the HiSeq or NextSeq platforms. A complete description of the genome sequencing analysis is detailed in the supplementary materials.

### Ago1p-Xrn1p co-immunoprecipitation

Overnight YPD cultures of haploid strains DPB215, DPB220, DPB221 were grown, diluted to OD_600_ 0.2 in 150 ml YPD, and then grown until OD_600_ 0.8. Cultures were harvested by centrifugation, washed with 25 ml cold phosphate-buffered saline (PBS), transferred to an Eppendorf tube in 750 μl cold PBS and resuspended in one volume IP buffer (50 mM HEPES pH 7.6, 300 mM NaCl, 5 mM MgCl_2_, 0.1 mM ethylenediaminetetraacetic acid (EDTA), 0.1 mM EGTA, 5% glycerol, 0% NP-40, PMSF with 2 cOmplete protease inhibitor cocktail tablets (Roche 04693116001) per 50 ml of buffer). Cells were lysed with acid-washed glass-bead beatings of 4 × 45 s with >4 min between bead beatings. The beads were removed via low-speed centrifugation, four volumes of IP buffer were added and cell debris was removed by centrifuging the extract at 21 000 × *g* for 5 s. The extract was cleared by centrifuging twice at 10 000 × *g* for 5 min and retaining the supernatant. Absorbance readings (260 nm) of 1:10 dilutions of the lysate were performed with a Nanodrop (Thermo Scientific). The amount of extract needed for 30 A260 units for each lysate was aliquoted, IP buffer was added to 990 μl and 10 μl RNase A/T1 cocktail (Ambion) or SUPERase-In was added. 0.3 A260 units of lysate was removed as the 1% input samples, the volume to was raised to 10 μl with IP buffer and then incubated at 4°C for 12 h. A total of 20 μl of EZview Red ANTI-FLAG M2 Affinity Gel (Sigma F2426) was added to lysates from strain DPB215 ± RNase and DPB221 or 20 μl of EZview Red Anti-HA Affinity Gel (Sigma E6779) was added to lysates from strain DPB215 ± RNase and DPB220. The lysates were incubated at 4°C for 12 h. The affinity agarose was sedimented by centrifugation, and the supernatant was removed. For the analysis to confirm RNA degradation, 3 A260 = 100 μl (10%) of supernatant was saved, and additionally 10 μl of the supernatant was saved for 1% supernatant samples. The affinity agarose was washed four times with IP buffer (each wash, 800 μl centrifuged 10 000 × *g* for 5 min), and the residual buffer was removed with a needle. A total of 100 μl 1× Laemmli buffer (Bio-Rad 1610737) + 2.5% beta-mercaptoethanol (bME) was added to the affinity agarose for each sample. A total of 10 μl 2× Laemmli buffer + 2.5% bME and 80 μl 1× Laemmli buffer + 2.5% bME was added to the input and supernatant samples. All samples were incubated at 90°C for 5 min, and then were centrifuged at room temperature for five min at 10 000 × *g*. All samples were run on a 4–12% Bis-Tris gel in MOPS buffer. The proteins were transferred from the gel to a PVDF membrane (Invitrogen LC2002), each blot was probed with anti-HA7 (1:2000) (Sigma H3663) and anti-Flag BioM2 (1:2000) (Sigma F9291) overnight at 4°C, and probed with the secondary antibody anti-mouse IgG HRP (1:5000) (Amersham NXA391) for 1–2 h at room temperature. The blots were stripped with 100 mM bME, 2% sodium dodecyl sulphate, 62.5 mM Tris pH 6.8 at 50°C for 30 min, shaking every 10 min. For the RNA-degradation analysis, samples containing 100 μl of supernatant were phenol extracted, precipitated and resuspended in 45 μl water. A total of 1 μl of the RNA sample was added to 2 μl water, and then 3 μl 2× glyoxal loading dye was added. Samples were then incubated at 50°C for 30 min to denature the RNA and then placed on ice. Samples were resolved on a 1% agarose gel run in NorthernMax-Gly Gel Prep/Running Buffer (Invitrogen AM8678) and visualized with a ChemiDoc MP Visualization System (Biorad).

### Ago1p, Dcp2p, Xrn1p *invivo* imaging

YPD cultures of strains DPB231 and DPB234 were grown overnight at 25°C. These overnight cultures were split in two, harvested by centrifugation, washed with CSM with or without glucose, resuspended in CSM with or without glucose and grown at 25°C for 1 h before imaging. About 488–543-nm lasers on a Zeiss LSM 510 confocal microscope were used to excite GFP and mCherry, respectively. Images were edited with ImageJ (49).

### Small-RNA sequencing, RNA-seq and analysis

Total RNA was isolated from *N. castellii* strains DPB220, DPB228, DPB622, DPB537 and DPB541. For small-RNA sequencing, 0.5 nM of four small RNA oligonucleotides (xtr miR-427, 5′-GAAAGUGCUUUCUGUUUUGGGCG; dme miR-14, 5′-GGGAGCGAGACGGGGACTCACT; Synthetic_siRNA_1_Guide, 5′-UAGUGCAGGUAGGUAUUUUUGUU; Synthetic_siRNA_1_Passenger, 5′-CAAAAAUACCUACCUGCACUAUA) were added to 10 μg of total RNA for use as internal standards. Small-RNA cDNA libraries were prepared as described (50), except 9–26-nt RNAs were isolated and sequenced, using 9-nt and 26-nt RNA radiolabeled standards (5′-AAACCAGUC and 5′ -AGCGUGUACUCCGAAGAGGAUCCAAA) to follow ligations and purifications.

For RNA-seq, 0.5 ng of two mRNAs (chloramphenicol and firefly luciferase) were added to 5 μg of total RNA for use as internal standards. Ribosomal RNA was depleted from the samples using Ribo-Zero Gold rRNA Removal Kit (Yeast) (Illumina, MRZY1324). RNA cDNA libraries were prepared using NEXTflex rapid directional mRNA-seq kit (Bioo Scientific, NOVA-5138-10). Libraries were sequenced using the Illumina SBS platform. Reads were mapped to the *N. castellii* genome, and those that mapped to small-RNA clusters (Supplementary Table S4) were fractionally counted. A complete description of the small-RNA sequencing and RNA-seq analysis is detailed in the Supplementary Materials.

### RNA and protein purification

A complete description of the purification of RNAs and proteins used in *invitro* assays is detailed in the Supplementary Materials.

### siRNA-binding assay

Proteins and RNAs were diluted in siRNA-binding buffer (50 mM Hepes pH 7.6, 1 mM EGTA, 1 mM EDTA, 5 mM magnesium acetate, 10% glycerol, 300 mM potassium glutamate, 10 mM NaCl, 0.01% NP-40, 5 mM DTT added fresh). About 10× RNA mix (10 nM miR-20a siRNA duplex with a 5′-radiolabeled guide strand, 100 nM unlabeled miR-20a siRNA duplex, 1 μM capped target RNA, 1 μM tRNA from baker's yeast (Sigma-Aldrich R5636), 1 μl SUPERase-In RNase Inhibitor (Invitrogen AM2696) per 20 μl RNA mix) was made, and 1.1 μl of this mix was added to a 1.5 ml G-tube siliconized microcentrifuge tube (BIO PLAS Inc., 4165SL). 1.1× protein mixes contained either no protein, 222 nM mut. Xrn1p, 222 nM WT Xrn1p, 111 nM WT Ago1p, 111 nM WT Ago1p and 222 nM mut. Xrn1p, or 111 nM WT Ago1p and 222 nM WT Xrn1p. Each protein mix was pre-incubated at 30°C for 5 min before adding 9.9 μl of the protein mix to the RNA mix. After incubation at 30°C for the indicated time, 10 μl of each reaction was removed and analyzed using a layered nitrocellulose–nylon filter-binding assay (51). Radiolabeled RNA was visualized by phosphorimaging (Fujifilm BAS-2500) and quantified using Multi Gauge (Fujifilm) software.

### Passenger-strand-cleavage and target-slicing assays

For both assays, proteins and RNAs were diluted in the siRNA-binding buffer. For the combined passenger-strand and target-slicing assay in Figure 4A–D, 5× RNA mix (5 nM miR-20a duplex with a 5′-radiolabeled passenger strand, 50 nM unlabeled miR-20a siRNA duplex; ∼500 nM cap-radiolabeled target RNA, 500 nM capped target RNA, 500 nM tRNA from baker's yeast and 1 μl SUPERase-In RNase Inhibitor per 20 μl RNA mix) was made, and 2 μl of this RNA mix was added to a 1.5 ml G-tube siliconized microcentrifuge tube. About 1.25× protein mixes contained either no protein, 250 nM mut. Xrn1p, 250 nM WT Xrn1p, 125 nM WT Ago1p, 125 nM WT Ago1p and 250 nM mut. Xrn1p, or 125 nM WT Ago1p and 250 nM WT Xrn1p. Each protein mix was pre-incubated at 30°C for 5 min before adding 8 μl of the protein mix to the RNA mix. After incubation at 30°C for the indicated time, 2 μl of the reaction was removed and quenched by adding to 5 μl of urea loading buffer (8M urea pH 8.0, 25 mM EDTA, 0.025% xylene cyanol, 0.025% bromophenol blue) and 3 μl water. For the assay of passenger-strand cleavage in Supplementary Figure S3A–C, the reactions were carried out as above, except no target was added to the RNA mix and the miR-20a siRNA duplex contained a 3′-cordycepin-labeled passenger strand.

For the Ago1p-RISC target-slicing assay in Figure 6A–C, 10× RNA mix (10 nM cap-radiolabeled target RNA, 1 μM capped target RNA, 1 μM tRNA from baker's yeast and 1 μl SUPERase-In RNase Inhibitor per 20 μl RNA mix) was made and 1 μl of this RNA mix was added to a 1.5 ml G-tube siliconized microcentrifuge tube. 1.1× protein mixes contained either no protein, 11.1 nM Ago1p-RISC, 11.1 nM Ago1p-RISC and 11.1 nM mut. Xrn1p, 11.1 nM Ago1p-RISC and 11.1 nM WT Xrn1p, 11.1 nM Ago1p-RISC and 33.3 nM mut. Xrn1p, 11.1 nM Ago1p-RISC and 33.3 nM WT Xrn1p, 11.1 nM Ago1p-RISC and 111.1 nM mut. Xrn1p, or 11.1 nM Ago1p-RISC and 111.1 nM WT Xrn1p. Each of the protein mixes was pre-incubated at 30°C for 20 min, before adding 9 μl of the protein mix to the RNA mix. After incubation at 30°C for the indicated time, 2 μl of each reaction was removed and quenched as for the combined assay. For the Ago1-RISC target-slicing assay in Figure 6D–F, the reactions were carried out as above, except the target was 3′-cordycepin-labeled.

To assess cleavage and slicing, RNAs were resolved on denaturing (7.5 M urea) 20% polyacrylamide gels, and radiolabeled cleavage products were visualized and quantified by phosphorimaging as above. At each time point, the fraction product was measured as *F*_p_ = product/(product + substrate). For Figure 6F, the fraction product was measured as *F*_p_ = product/(product + substrate + degradation intermediate). For Figure 6C, the multiple-turnover slicing data were fit in Prism 8 (GraphPad) to the burst and steady-state equation (52,53),

$$\begin{equation*} F \left( t \right) = E \times \frac{{{a^2}}}{{{{\left( {a + b} \right)}^2}}}\left( {1 - {e^{ - \left( {a + b} \right)t}}} \right) + E \times \frac{{ab}}{{a + b}}t, \end{equation*}$$

Where *F(t)* is target cleaved over time, *E* is the enzyme concentration, and *a* and *b* are rate constants (reported as *k*_1_ and *k*_2_, respectively) according to the following scheme,

$$\begin{equation*} E + S\mathop \to \limits^a \ E \cdot P\ \mathop \to \limits^b \ E + P. \end{equation*}$$

## RESULTS

### *XRN1* is a component of the budding-yeast RNAi pathway

A genetic selection was designed to discover novel components of the RNAi pathway in *N. castellii*. This selection centered on an *N. castellii* strain that expressed RNA hairpins that were processed by Dicer to produce siRNAs targeting *HIS3*, *URA3* and *GFP* mRNAs expressed from exogenous genes that had been integrated into the genome (Figure 1A). The hairpin and *GFP* genes were under the control of the *S. cerevisiae GAL1* and *URA3* promoters, respectively, and the *HIS3* and *URA3* genes, together with their promoters, were obtained from *S. cerevisiae* and replaced their *N. castellii* counterparts in the genome of the selection strain. Because this parental strain was engineered to deploy the RNAi pathway to silence *HIS3* and *URA3*, it did not grow on media lacking histidine and uracil. This strain was mutagenized with ethyl methane sulfonate (EMS), and the cells were plated on media lacking histidine and uracil with the goal of identifying mutants that had a His+ and Ura+ phenotype because they no longer efficiently silenced *HIS3* and *URA3*. As a secondary screen, mutants were examined by flow cytometry for their ability to silence *GFP*, and those that had impaired *GFP* silencing were carried forward for complementation analysis (Figure 1A).

Analysis of mutants isolated in pilot experiments illustrated the ability of our selection to find mutants in the RNAi pathway. Mutant strains from these pilot selections were mated to each of three strains: WT, Δ*ago1* and Δ*dcr1*, and the resulting diploids were assessed for their ability to silence *GFP* (Figure 1A). If a new mutation was recessive and in the *AGO1* gene, then the diploid formed with Δ*ago1* would fail to silence *GFP*, and likewise, if a new mutation was recessive and in the *DCR1* gene, then the diploid formed with Δ*dcr1* would fail to silence *GFP*. Indeed, these complementation analyses indicated that all of the strains tested had mutations in either *AGO1* or *DCR1*, which showed that the selection scheme worked. To identify additional genes involved in RNAi without also having to contend with a large number of *ago1* and *dcr1* mutants, we modified the selection strain to contain an extra copy of *AGO1* and *DCR1*, each under control of its native promoter, integrated into the genome at an ectopic location.

Mutations that lost silencing were selected in the new parental selection strain (DPB537) with duplicated *AGO1* and *DCR1* genes. As expected, isolated mutant strains but not the parental strain grew on selective media, as assessed by a serial-dilution growth assay (Figure 1B). Each of the isolated strains carried recessive mutations, as assessed from analyses of diploid products of mating with a WT strain. Moreover, as illustrated for strain 3 (Figure 1A), 19 strains retained both Ago1p and Dcr1p activities, as indicated by fully restored *GFP* silencing in diploid products of both Δ*ago1* and Δ*dcr1* matings. These 19 strains were carried forward as potentially having mutations in genes encoding another protein needed for efficient RNAi.

To identify mutated genes, we sequenced the genomes of the parental and mutant strains (130-fold and 65- to 248-fold coverage, respectively), which identified many protein-coding or tRNA changes in each of the mutant genomes (median 47, range 2–120) (Supplementary Table S1). Of the 19 mutant strains sequenced, seven had a mutation in *XRN1*, the gene for the 5′-to-3′ cytoplasmic exoribonuclease that is highly conserved in eukaryotes (Supplementary Table S2). All seven were either G-to-A or C-to-T transition mutations characteristic of EMS mutagenesis, and six of the seven were nonsense mutations. The second most frequently mutated gene was *SRB8*, a component of the mediator complex (54,55), which was mutated in four strains, the mutations in three being transitions and the fourth being a single-base-pair deletion. Two strains had mutations in both *XRN1* and *SRB8*.

To examine the ability of *XRN1* and *SRB8* mutations to confer growth on selective media, we tested the growth of the parental selection strain with single and double knockouts of these genes. The *Δxrn1 Δsrb8* strain grew on selective media, whereas *Δxrn1* or *Δsrb8* mutant strains failed to grow (Figure 1C). The requirement for the *SRB8* mutation can be attributed to the fact that the hairpins used to silence the *HIS3*, *URA3* and *GFP* genes were each expressed from the *GAL1* promoter. Any mutation that lowered transcription from the *GAL1* promoter would decrease silencing and increase expression of the *HIS3*, *URA3* and *GFP* genes. A mutation in *SRB8*, as a subunit of the mediator complex, might have provided that reduced hairpin expression. Supporting this idea, *SRB8* is transcriptionally induced in the presence of galactose, as are *SSN2* and *SSN3*, two of the other genes that were disrupted in multiple mutant strains, including strains with *XRN1* mutations (54,56–57). Taken together, these observations suggested that a mutation in *SRB8* (or in either *SSN2* or *SSN3*) created a permissive background, in the context of which a mutation in *XRN1* imparted growth. In this scenario, *XRN1* mutations presumably reduced the efficiency of the RNAi pathway without fully disrupting it.

To test the possibility that loss of *XRN1* function leads to a partial reduction in RNAi efficiency, we determined the ability of *XRN1* mutations to silence *GFP*. Seven haploid *xrn1*-mutant strains from our selection were mated with a haploid Δ*xrn1* strain, and we assessed the ability of the resulting diploid strains to silence *GFP*. The experiment compared the silencing of *XRN1/XRN1*, *XRN1/*Δ*xrn1*, Δ*xrn1*/Δ*xrn1* with Δ*xrn1*/*xrn1*-1-7. Six of these seven diploid strains had impaired *GFP* silencing (Figure 1D). These six each derived from a mutant strain with a nonsense mutation in *XRN1*, whereas the one with efficient *GFP* silencing derived from the strain with a missense mutation in *XRN1* (RNAi mutant 5). These results indicated that the missense mutation probably did not impair Xrn1p activity but that the *XRN1* nonsense mutations were each loss-of-function mutations and were at least partially responsible for the reduced *GFP* silencing observed in the haploid mutant strains. Indeed, the Δ*xrn1*/Δ*xrn1* diploid that we constructed, which had no other confounding mutations, also had reduced silencing.

We also examined the consequence of inactivating *XRN1* in an RNAi-reporter strain containing an integrated *GFP* gene and an integrated gene expressing stem-loop transcripts that can be processed into siRNAs that target *GFP*. Compared to deleting *AGO1* or *DCR1*, deleting *XRN1* produced an intermediate *GFP*-silencing defect (Figure 1E), which depended on the presence of the silencing construct (Supplementary Figure S1). The observation that silencing was reduced but not completely abolished supported the conclusion that Xrn1p functions in the budding-yeast RNAi pathway but, unlike Dcr1p and Ago1p, is not an essential component of the pathway. In contrast, disrupting other genes involved in cytoplasmic mRNA decay, including *DCP2* or *SKI3*, which code for proteins important for decapping and 3′-to-5′ degradation, respectively (58,59), had minimal effect on silencing *GFP* (Figure 1E), indicating that their respective decay activities are not required for efficient RNAi in budding yeast.

### Xrn1p and Ago1p physically interact and colocalize

**Figure 2.**
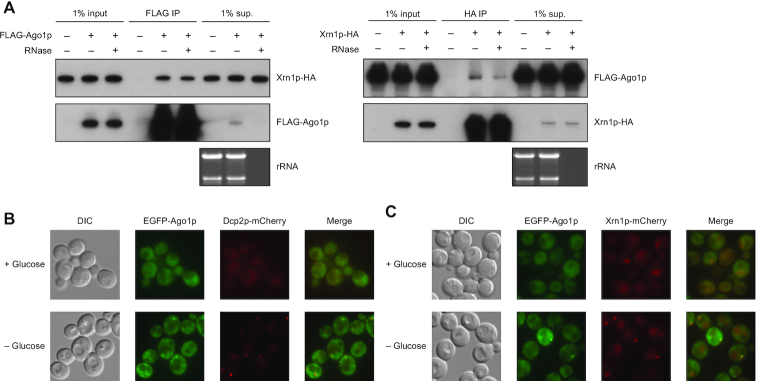
Physical interaction and colocalization of Xrn1p and Ago1p in *Naumovozyma castellii*. (**A**) Physical interaction of Xrn1p and Ago1p in *N. castellii*. Immunoblots show the results of co-immunoprecipitations of FLAG-tagged Ago1p (FLAG-Ago1p, left) and HA-tagged Xrn1p (Xrn1p-HA, right). The samples on the blots were from 1% of the input into the immunoprecipitation (1% input), the immunoprecipitation with anti-FLAG or anti-HA antibodies (FLAG-IP and HA-IP, respectively) and 1% of the remaining supernatant after immunoprecipitation (1% sup.). The immunoprecipitations were performed with and without treatment with an RNase A/T1 cocktail. (**B**) Co-localization of Ago1p and Dcp2p observed in *N. castellii* grown in the presence and absence of glucose. Fluorescence microscopy images show the location of EGFP-tagged Ago1p and mCherry-tagged Dcp2p. (**C**) Co-localization of Ago1p and Xrn1p observed in *N. castellii* grown in the presence and absence of glucose. Fluorescence microscopy images show the location of EGFP-tagged Ago1p and mCherry-tagged Xrn1p.

As an orthogonal approach to identify components of the budding-yeast RNAi pathway, we expressed epitope-tagged Ago1p in *N. castellii* and used mass spectrometry to identify co-immunoprecipitating proteins. Xrn1p was the most significantly enriched protein in both biological replicates of this experiment (Supplementary Table S3), suggesting that Xrn1p physically interacts with Ago1p in these cells. This physical interaction was supported by reciprocal co-immunoprecipitation of Ago1p and Xrn1p as assayed by immunoblotting (Figure 2A). Co-immunoprecipitation was at least partially retained in the presence of RNase, which indicated a protein–protein interaction (Figure 2A).

To examine the localization of Ago1p and Xrn1p in *N. castellii* cells, we tagged the proteins with GFP and mCherry, respectively. In *S. cerevisiae* grown under standard conditions, Xrn1p is reported to be diffusely cytoplasmic, but upon glucose starvation, it preferentially localizes to P-bodies, along with other known RNA-decay components including Dcp2p (60). In *N. castellii*, we observed similar behavior of diffuse localization and P-body localization in normal conditions and glucose starvation, respectively, for not only Xrn1p but also Ago1p (Figure 2B and C). The colocalization of Ago1p and Xrn1p in *N. castellii* further supported the conclusion that Xrn1p plays a role in RNAi.

### *XRN1* affects small RNA populations in budding yeast

**Figure 3.**
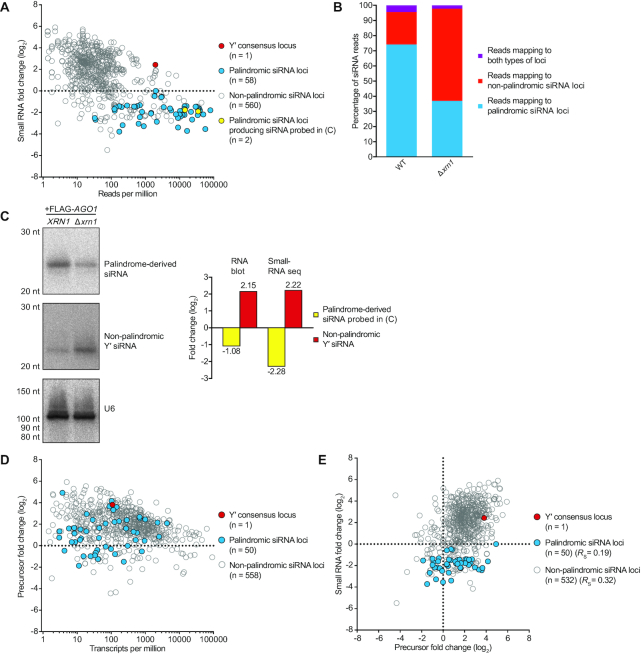
The effect of Xrn1p on the abundance of small RNAs and their precursors in *Naumovozyma castellii*. (**A**) The effect of Xrn1p on the abundance of small RNAs originating from siRNA-producing loci yielding at least 1 RPM (read per million) in WT cells. The scatter plot shows the fold change (log_2_) of 22–23-nt small RNAs deriving from each locus in the Δ*xrn1* versus WT strain (after normalizing to the recovery of internal standards) as a function of the abundance of 22–23-nt small RNAs deriving from the locus in the WT strain. The Y′ consensus locus is represented by the filled red circle, and other non-palindromic loci are represented by empty gray circles. The two palindromic loci probed in C are represented by the filled yellow circles, and the other palindromic loci are represented by the filled cyan circles. For each category, the number of loci is indicated in parentheses. Sequencing was from strains in which the endogenous Ago1p had been FLAG tagged. (**B**) Overall effect of Xrn1p on the fraction of palindrome-derived and non-palindrome-derived small-RNA populations in *N. castellii*. The 22–23-nt reads that mapped to siRNA-producing loci were designated as deriving from either palindromic loci (cyan), non-palindromic loci (red), or both types of loci (purple) in the same strains as A. (**C**) Effect of Xrn1p on the expression levels of both a palindrome-derived siRNA and a non-palindrome-derived siRNA in *N. castellii*. At the left are phosphorimages of a small-RNA blot successively probed for siRNAs originating from either palindromic or non-palindromic precursor transcripts in the same WT and Δ*xrn1* strains as A. The migration of markers is indicated on the left. For a loading control, the blots were re-probed for the U6 snRNA. To the right of the blots is a bar graph showing the effect of deleting *XRN1* on these siRNAs, as measured both by RNA blots and by the sequencing in A. (**D**) Effect of Xrn1p on the abundance of small-RNA precursor transcripts in *N. castellii*, as measured using RNA-seq. Shown for each precursor transcript expressed at ≥1 transcript per million (TPM) in WT cells is its fold change (log_2_) in the Δ*xrn1* versus WT strains (after normalizing to the recovery of internal standards) as a function of its abundance in WT cells. Strains were as in A, and precursor transcripts are classified as in A. (**E**) Relationship between changes in small RNAs and changes in siRNA precursors observed after deleting *XRN1*. Shown for each precursor transcript expressed at ≥1 TPM in WT cells and that produced small RNAs at ≥1 RPM is the fold change (log_2_) in 22–23-nt small RNA reads observed in A as a function of the fold change (log_2_) in precursor transcripts observed in D. Representation of each type of locus as in A. The number of loci and Spearman's rank correlation coefficient (*R*_s_) for each category is indicated in parentheses.

To learn whether Xrn1p impacts the levels of siRNAs in the cell, we sequenced small RNAs from WT and Δ*xrn1 N. castellii* strains after adding known quantities of synthetic standards to each sample, which enabled quantitative comparisons between samples. Mapping of the 22–23-nt reads to previously identified siRNA-producing loci (Supplementary Table S4) (8) and normalizing to the synthetic standards showed that siRNA levels from the most prolific loci were reduced upon *XRN1* disruption (Figure 3A). Many of these prolific loci were inverted repeats (i.e. palindromic loci), which produce transcripts with a hairpin (stem-loop) structure. For nearly all of the palindromic loci, fewer siRNAs accumulated in the Δ*xrn1* strain (median change, 3.5-fold) (Figure 3A). In contrast, siRNAs mapping to Y′ subtelomeric elements and other non-palindromic loci, in which convergent overlapping transcription produces transcripts that base pair to form bimolecular siRNA precursor duplexes, typically increased in the Δ*xrn1* strain (median change, 4.1-fold). For example, siRNAs from the Y′ subtelomeric elements increased by over 5-fold in the *XRN1*-disrupted strain (Figure 3A and Supplementary Figure S2B). Subtelomeric siRNAs also accumulate in *S. pombe* upon the deletion of its *XRN1* ortholog, *exo2* (61). When classifying the 22–23-nt reads as originating from either palindromic or non-palindromic loci, the proportion of siRNA reads originating from palindromic loci decreased by half in the *XRN1*-disrupted strain (from 74.1 to 37.0%) and the siRNA reads that mapped to non-palindromic loci increased (from 21.5 to 60.8%) (Figure 3B). The disparate changes for the different types of siRNAs observed by sequencing were also observed by probing an RNA blot analyzing small RNAs from the same strains (Figure 3C). This blot showed that an siRNA deriving from a palindromic region decreased by > 2-fold in the Δ*xrn1* strain, whereas a Y′ subtelomeric siRNA increased in expression by > 4-fold. The Y′ subtelomeric siRNA also increased in Δ*dcp2* and Δ*ski3* strains but not to the same degree as in the Δ*xrn1* strain, whereas the palindromic siRNA increased in the Δ*ski3* strain and did not substantially change in the Δ*dcp2* strain (Supplementary Figure S2A).

To ascertain if changes in siRNAs observed in the Δ*xrn1* strain could be attributed to changed accumulation of their siRNA-precursor transcripts, we performed RNA sequencing (RNA-seq) of longer transcripts, again mapping sequencing reads to annotated siRNA-producing loci. Many siRNA-precursor transcripts increased in expression in the Δ*xrn1* strain (Figure 3D, median change, 2.2- and 3.8-fold for palindromic and non-palindromic loci, respectively). For instance, the Y′ subtelomeric siRNA precursor transcripts increased by over 14-fold in the *XRN1*-disrupted strain (Figure 3D and Supplementary Figure S2B). Examination of the relationship between changes in siRNAs and changes in precursor transcripts uncovered a modest correlation for products of non-palindromic loci (*R*_S_ = 0.32; *P* < 0.001), with increased levels observed for both siRNAs and precursors from 430 of 532 loci (Figure 3E). In contrast, little correlation was observed for products of palindromic loci (*R*_S_ = 0.19; *P =* 0.2).

Similar expression changes of small RNAs and their precursor transcripts were observed when comparing the *N. castellii* selection strain and a Δ*xrn1* strain derived from it (Supplementary Figure S2C and D). In these strains, the most abundant siRNAs mapped to the loci expressing hairpins corresponding to the exogenous *HIS3*, *URA3* and *GFP* genes. The siRNAs from these three loci decreased by 2.7-, 1.8- and 1.1-fold, respectively, in the Δ*xrn1* strain (Supplementary Figure S2C). The precursor transcripts for both the endogenous palindromic and non-palindromic siRNAs also increased in the Δ*xrn1* selection strain (Supplementary Figure S2D, median fold change of 3.5 and 4.6, respectively). As with the non-selection strains, when examining the relationship between the changes in siRNAs and the changes in precursor transcripts, a modest correlation was observed for products of non-palindromic loci (Supplementary Figure S2E, *R*_S_ = 0.33; *P* < 0.001), and little correlation was observed for products of palindromic loci (Supplementary Figure S2E, *R*_S_ = 0.04; *P =* 0.38).

These results suggest that the less efficient silencing of *GFP* observed in the Δ*xrn1* strain was likely at least partially attributable to reduced siRNAs deriving from the palindromic transcripts designed to silence *GFP*. One possibility is that the loss of *XRN1* causes an increase of non-palindromic siRNA precursors, and the increased siRNAs deriving from these precursors compete with the palindrome-derived siRNAs for loading into Ago1p, thereby causing more of the *GFP* siRNAs to be degraded before loading into Ago1p. In this scenario, Xrn1p could influence the levels of non-palindromic siRNA precursors either directly, by degrading these precursors, or indirectly, by increasing the efficiency of RNAi-mediated degradation of these transcripts, which are targets of as well as precursors for siRNAs of the RNAi pathway.

### Xrn1p enhances RNAi *invitro*

**Figure 4.**
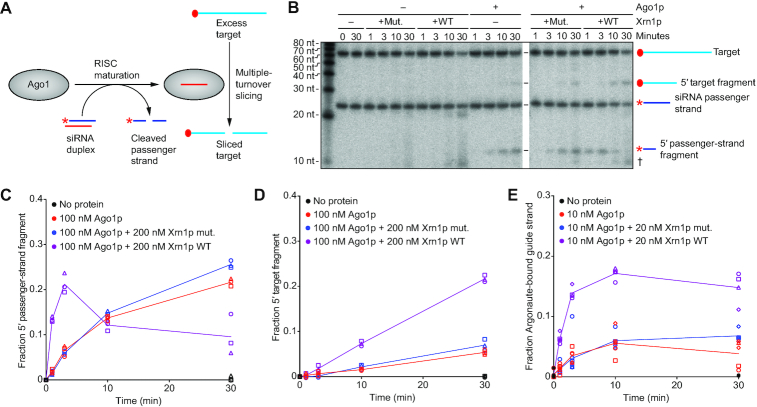
The impact of Xrn1p on siRNA loading, passenger-strand degradation and target slicing. (**A**) Experimental scheme of a combined assay monitoring both passenger-strand cleavage and target slicing. An siRNA duplex is loaded into Ago1p, which cleaves and discards the passenger strand before beginning to catalyze multiple-turnover slicing of the target RNA. Initial concentrations of siRNA duplex and target RNA were 10 and 100 nM, respectively. In the duplex, the red star indicates radiolabeled monophosphate on the 5′-end of the passenger strand. On the target, the red-filled circle indicates a 5′-radiolabeled cap. (**B**) The effect of Xrn1p on Ago1p-catalyzed passenger-strand cleavage and target slicing, in the assay schematized in A. Shown is a representative denaturing gel that resolved cap-labeled target, cap-labeled target fragment, 5′ end-labeled passenger strand and 5′ end-labeled passenger-strand fragment after incubation for the indicated time, with or without purified Ago1p (100 nM) and with or without purified Xrn1p (WT or mutant [mut.]) (200 nM). The cross symbol (†) indicates degradation of the target after adding Xrn1p, which was observed at late time points and presumed to be due to a contaminant in the Xrn1p preparation. (**C**) Quantification of the passenger-strand cleavage. Results are shown for three replicates (circles, squares, triangles) of reactions with either no protein (black), Ago1p only (red), Ago1p with Xrn1p mut. (blue), or Ago1p with Xrn1p WT (purple). The fraction of passenger-strand fragment was calculated by dividing the signal of product/(product + substrate). The lines, added for clarity, connect mean values and are not fit to an equation. (**D**) Quantification of the 5′ target fragment. Otherwise, as in C. (**E**) The effect of Xrn1p on siRNA association with Ago1p. Shown are results of a filter-binding assay that measured association of 5′ radiolabeled guide RNA with Ago1p after incubating components of the combined assay of passenger-strand cleavage and target slicing. Each assay included siRNA duplex, unlabeled target RNA and the other components indicated, each at the same concentrations used in B. Results are shown for five independent experiments (circles, squares, triangles, diamonds, hexagons), which together contributed at least three data points for each time point for reactions with either no protein (black filled circles), Ago1p only (red), Ago1p and Xrn1p mut. (blue), or Ago1p and Xrn1p WT (purple). The fraction of Ago1p-bound guide strand was calculated by dividing the signal for the RNA that bound to the nitrocellulose membrane by that of the RNA that bound to the nylon and nitrocellulose membranes combined.

To study the ability of Xrn1p to enhance RNAi, we purified WT *N. castellii* Ago1p and WT and catalytically impaired versions of *N. castellii* Xrn1p (each from *S. cerevisiae* overexpression strains that lacked *XRN1*) and examined the influence of Xrn1p activity on passenger-strand cleavage and target slicing *invitro*. The *invitro* assay used an siRNA duplex with a 5′-labeled passenger strand and a cap-labeled target RNA to follow both passenger-strand cleavage and target slicing in multiple-turnover conditions (Figure 4A). In the presence of WT but not catalytically impaired (mut.) Xrn1p, the amount of passenger-strand cleavage fragment substantially increased at early time points and then dropped at later time points (Figure 4B and C). The reduced passenger-strand fragment observed at later time points indicated that Xrn1p degraded this fragment, either as it exited RISC or after it dissociated from RISC—in either case, potentially enhancing RNAi efficiency by facilitating RISC maturation or preventing cleaved passenger strand from inhibiting RISC activity, respectively. An siRNA duplex with a 3′-labeled passenger strand showed a similar trend as the siRNA duplex with a 5′-labeled passenger strand; in the reaction with WT Xrn1p, the 3′-cleavage fragment of the passenger strand accumulated to a greater extent at early time points and then was degraded at later time points (Supplementary Figure S3A–C). These results indicated that Xrn1p degrades both cleavage fragments of the passenger strand *invitro*—an observation consistent with the known preference of Xrn1p for substrates with a 5′ monophosphate.

WT Xrn1p also increased the rate of target slicing (Figure 4D). The increased rates of both passenger-strand cleavage and target slicing suggested that Xrn1p increased the amount of Ago1p that could be loaded with an siRNA duplex, perhaps by degrading non-specifically bound cellular RNAs that might be occluding siRNA loading. To assess whether Xrn1p activity increased the amount of Ago1p that could be loaded with a siRNA duplex, we performed filter-binding assays to measure the amount of siRNA bound to protein after incubating guide-labeled siRNA duplex with the purified proteins (Figure 4E). Preincubation of WT Xrn1p with Ago1p enhanced the amount of siRNA bound to protein, whereas preincubation with mutant Xrn1p did not have this effect (Figure 4E). These results supported the idea that the exonuclease activity of Xrn1p might help eliminate non-siRNA species that spuriously bind Ago1p and competitively inhibit siRNA duplex binding.

### *XRN1* affects the stability of the passenger strand *invivo*

**Figure 5.**
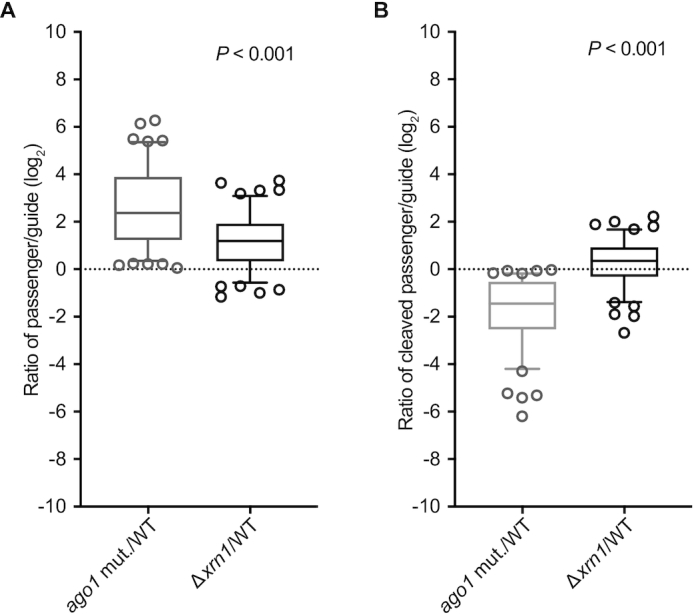
The influence of Xrn1p on the accumulation of the siRNA passenger strand and its cleavage fragments *invivo*. (**A**) Accumulation of full-length passenger strands. Box-and-whisker plots show the log_2_ ratios of the reads for the full-length passenger strand over the reads for the full-length guide strand in the slicing-impaired *ago1* mutant (mut.) versus WT strains and in Δ*xrn1* versus WT strains. Results from 114 guide–passenger duplexes are plotted, with the line indicating the median, the box indicating 25–75 percentiles, the whiskers indicating the 5–95 percentiles and open circles indicating individual outliers. The two-tailed Wilcoxon signed-ranked test was used to evaluate the similarity of the two distributions (*P*-value). (**B**) Accumulation of cleaved passenger strands. Box-and-whisker plots show the log_2_ ratios of the reads for the cleaved passenger strand over the reads for the full-length guide strand in *ago1* mut. versus WT strains and in Δ*xrn1* versus WT strains. Otherwise, as in A.

Because *N. castellii* Xrn1p degraded the cleaved passenger strand *invitro*, we asked if it had this effect *invivo*. We identified perfectly paired guide–passenger duplexes for which 22–23-nt reads were observed among small-RNA reads from WT, Δ*xrn1* and *AGO1* slicing-impaired (*ago1*-*D1247R*) *N. castellii* strains. For each of these duplexes, the strand with more reads in the WT strain was designated as the guide, the other strand was designated as the passenger and duplexes for which the passenger-to-guide ratio increased in the *ago1* mutant strain were carried forward as experimentally supported siRNA duplexes. Twelve-nucleotide reads that perfectly matched the 3′ end of the passenger strand were designated as 3′-cleavage fragments of passenger strands, and depending on whether the duplex was comprised of 22- or 23-nt species, 10- or 11-nt reads that perfectly matched the 5′ end of the passenger strand were respectively designated as 5′-cleavage fragments of passenger strands. For most duplexes, disrupting *XRN1* increased the ratio of full-length passenger strand to guide strand, as would be expected if Xrn1p facilitates RISC loading (Figure 5A, median increase, 2.3-fold; *P* < 0.001, Wilcoxon signed-rank test). Disrupting *XRN1* also increased the ratio of cleaved passenger strand to guide strand modestly (Figure 5B, median increase, 1.3-fold; *P* < 0.001).

We also examined the length distributions and the first nucleotide composition of the genomically mapping 9–26-nt reads in the libraries of each of these strains, excluding reads that mapped exclusively to rRNAs or tRNAs (Supplementary Figure S4). There were no substantial differences in the size distributions of the 9–26-nt reads in the Δ*xrn1* or *AGO1* slicing-impaired strains compared with the WT strain, although there were slightly more 15–20-nt reads in the Δ*xrn1* strain (Supplementary Figure S4). Similar to previous studies, most 23-nt RNAs began with U, which suggested that most siRNA guide strands begin with this nucleotide (8,62). The Δ*xrn1* and *AGO1* slicing-impaired strains had a higher fraction of 23-nt reads with A in the third to last position (24.8 and 36.0%, respectively, compared to 19.3% for the WT strain), which would be expected of full-length passenger strands that could pair with guide strands that begin with U. These results indicated that Xrn1p affects the stability of full-length and cleaved passenger strands *invivo*, as it does *invitro*.

### Xrn1p enhances target slicing and eliminates the 3′ slicing fragment

**Figure 6.**
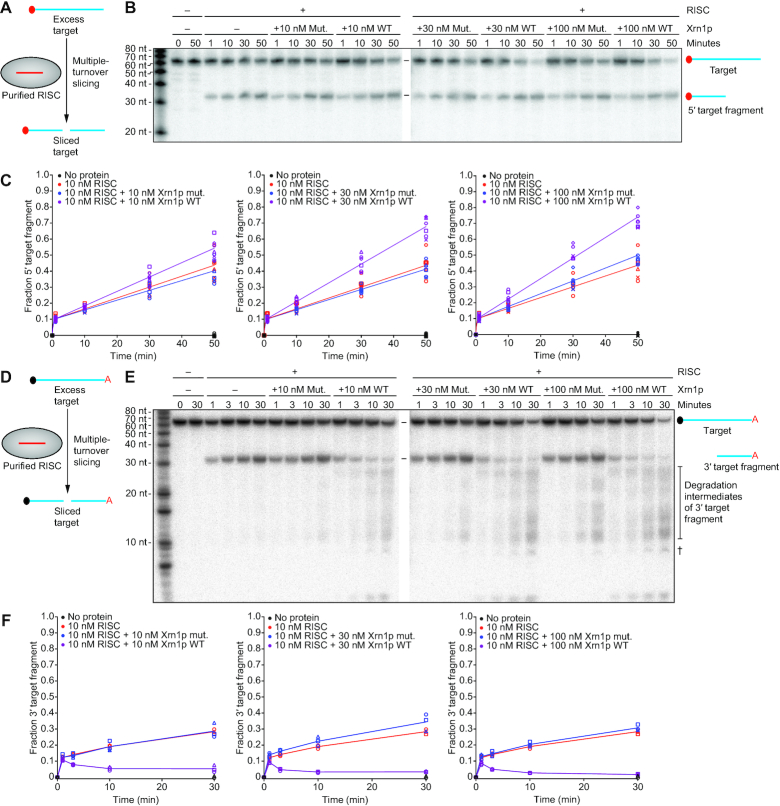
The impact of Xrn1p on multiple-turnover slicing and on stability of the 3′ target fragment. (**A**) Experimental scheme of the assay for multiple-turnover slicing using a cap-labeled target. The red-filled circle on the target RNA indicates a radiolabeled 5′ cap. The initial concentration of target was 100 nM. (**B**) The effect of Xrn1p on multiple-turnover slicing in the assay schematized in A. Shown is a representative denaturing gel resolving target from its sliced product after incubation with or without purified RISC–miR-20a (10 nM), with the indicated concentration of Xrn1p (WT or mut.; 0, 10, 30 or 100 nM) for the indicated amount of time. (**C**) Quantification of the 5′ product of target slicing. Results are shown for six independent experiments (circles, squares, triangles, diamonds, hexagons and exes) of reactions with either no protein (black), RISC–miR-20a only (red), RISC–miR-20a with Xrn1p mut. (blue), or RISC–miR-20a with Xrn1p WT (purple). The fraction of 5′ target fragment was calculated by dividing the signal of product/(product + substrate). The lines indicate the best fit to the burst equation. (**D**) Experimental scheme of the assay for multiple-turnover slicing with a 5′-capped, 3′-radiolabeled target. The black-filled circle on the target indicates a 5′-cap, and the red A indicates a radiolabeled cordycepin at the 3′-end of the target. The initial concentration of target was 100 nM. (**E**) The effect of Xrn1p on multiple-turnover slicing in the assay schematized in D. The cross symbol (†) indicates degradation of the target after adding Xrn1p, which was observed at late time points and presumed to be due to a contaminant in the Xrn1p preparation. Otherwise, as in B. (**F**) Quantification of the 3′ product of target slicing. Results are shown for three independent experiments (circles, squares, triangles). The lines, shown for clarity, connect mean values and are not fit to an equation. Otherwise, as in C.

To learn whether Xrn1p enhances the efficiency of any steps following duplex loading, RISC maturation and passenger-strand degradation, we purified mature *N. castellii* RISC programmed with a specific guide RNA (63) and examined the ability of *N. castellii* Xrn1p to enhance slicing of cap-labeled target *invitro*. Under single-turnover conditions, in which the RISC enzyme was in excess over target, Xrn1p activity had no detectable influence on slicing rates (Supplementary Figure S5A and B). Likewise, Xrn1p activity had no detectable influence on the initial burst phase of a multiple-turnover slicing reaction (Figure 6A), in which target was in 10-fold excess over the RISC enzyme (Figure 6B and C). However, after the slicing product reached the concentration of RISC (10 nM) and the reaction entered a slower phase characterized by rate-limiting product release, WT Xrn1p but not the catalytically impaired Xrn1p increased the rate of target slicing (Figure 6B and C). Enhancement of this second phase of target slicing increased after boosting the Xrn1p concentration from 10 to 30 nM but did not increase with even more Xrn1p (Figure 6C).

These results suggested that Xrn1p enhances release of sliced product, presumably by degrading the 3′ product of slicing, which contains the 5′-monophosphate characteristic of Xrn1p substrates. To examine whether Xrn1p has this function, we performed a slicing reaction with a 5′-capped, 3′-labeled target RNA in multiple turnover conditions (Figure 6D). In reactions lacking Xrn1p or containing catalytically impaired Xrn1p, the 3′ slicing product accumulated rapidly during the burst phase and then more slowly during the part of the reaction characterized by rate-limiting product release, as observed for the 5′ product in reactions with cap-labeled target (Figure 6E and F). In reactions containing WT Xrn1p, the 3′ slicing product similarly accumulated at an early time point but then began to decrease as it was degraded by Xrn1p (Figure 6E and F). This rate of degradation increased as the concentration of Xrn1p increased from 10 to 30 nM but did not significantly increase with additional Xrn1p (Figure 6F). The elimination of this 3′ product of slicing, which pairs to the guide-RNA seed region, which in turn is critical for target recognition and binding (52,64–65), would prevent this 3′ slicing product from rebinding to the guide and would thereby allow RISC to bind and slice another target RNA.

In support of the proposal that Xrn1p degrades 3′ products of slicing, comparison of RNA-seq data from the selection strain with and without *XRN1* showed that, in the absence of Xrn1p, RNA preferentially accumulated in the mRNA regions downstream of *HIS3*, *URA3* and *GFP* slicing (Supplementary Figure S5C). In contrast, endogenous genes that were not targets of RNAi in the selection strain showed a uniform increase in RNA sequencing reads mapping across the gene in the *XRN1-*knockout strain, and showed an increased proportion of reads mapping to their 3′ ends in both WT and *XRN1*-deficient backgrounds (Supplementary Figure S5D). Thus, as observed in other species (30–32), *XRN1* is responsible for eliminating the 3′ products of slicing in budding yeast.

## DISCUSSION

**Figure 7.**
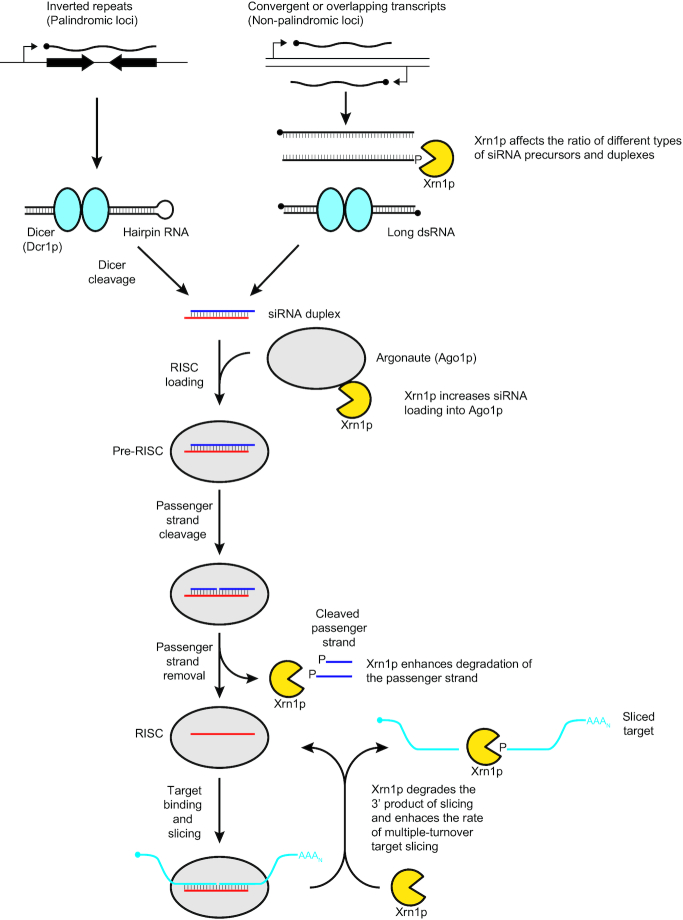
Schematic depicting the roles of Xrn1p in budding yeast RNAi. See main text for description.

Our results show that Xrn1p influences most steps of the RNAi pathway in budding yeast (Figure 7). Xrn1p increases the levels of siRNAs that accumulate from palindromic loci, presumably by reducing the amount of non-palindromic siRNA-precursor transcripts, thereby reducing competition for entry into the pathway from siRNAs that would otherwise be produced from these non-palindromic precursors. Once the siRNA duplexes are produced, Xrn1p facilitates their loading into Argonaute, perhaps by eliminating other RNA species that spuriously bind to Argonaute and thereby inhibit duplex loading. After duplexes bind to Argonaute and passenger strands are cleaved, Xrn1p helps degrade the cleavage fragments, which prevents inhibition of RISC and might facilitate its maturation. Finally, Xrn1p facilitates multiple turnover of RISC by eliminating the 3′ products of slicing, thereby preventing product inhibition and perhaps helping to regenerate unbound RISC. We also detected a physical interaction between Ago1p and Xrn1p, which might play a role in one or more of these functions.

Even with these new-found roles of Xrn1p, the RNAi pathway of budding yeast appears to be the most streamlined of any characterized to date, with only three known components: Dcr1p, Ago1p and Xrn1p. Although we cannot rule out participation of other factors that might have been missed by our genetic and pulldown approaches, the notion that RNAi in budding yeast relies on only three proteins seems plausible. For example, the most notable difference between budding-yeast Ago1p and Argonaute proteins characterized from other lineages is that purified Ago1p can autonomously load an siRNA duplex and then cleave and remove the passenger strand of this duplex to form active RISC capable of slicing target RNA (66), whereas other Argonaute proteins require the Hsc70/Hsp90 chaperone and adenosine triphosphate to assume a conformation capable of duplex loading and RISC maturation (19–21,23–24). That Ago1p can autonomously perform these functions *invitro*, with some enhancement by Xrn1p, suggests that other factors might not be required for duplex loading and RISC maturation in budding yeast.

The unusual domain structure and activity of budding-yeast Dcr1p might also obviate the need for other accessory factors needed for siRNA loading into Argonaute in other systems. Budding-yeast Dcr1p possesses only one RNase III domain, whereas canonical Dicers possess two RNase III domains (67). Consistent with RNase III domains always functioning in pairs (68), budding-yeast Dicer forms a homodimer to cleave both strands of the dsRNA (67). Whereas canonical Dicers measure from the end of the dsRNA to determine the site of cleavage (69,70), multiple Dcr1p homodimers bind cooperatively within the dsRNA, with the distance between active sites of adjacent homodimers determining the length of the siRNA duplexes (67). Each unit of the budding-yeast homodimer possesses two dsRNA-binding domains (dsRBDs), which are both required for accumulation of siRNAs to normal levels *invivo*, although the second dsRBD is dispensable for producing siRNAs *invitro* (67). One proposal is that in cells this second dsRBD acts after siRNA production, in tandem with the second dsRBD contributed by the other Dcr1p of the homodimer, to perform functions normally provided by dsRBD-containing Dicer accessory factors, such as helping to transfer the siRNA duplex from Dicer to Argonaute, thereby obviating the need for these cofactors (67).

Further supporting the existence of a streamlined pathway in budding yeast, *N. castellii* lacks orthologs of other factors that participate in RNAi and related pathways in other species. For example, *N. castellii* and all other RNAi-possessing budding-yeast species lack recognizable homologs of RdRPs (8). Budding yeast also lack a discernible ortholog of *HEN1*, which methylates the 2′ oxygen of the 3′-terminal nucleotide of plant and metazoan siRNAs, plant miRNAs, as well as metazoan Piwi-interacting RNAs (piRNAs), thereby protecting these small RNAs from 3′-end uridylation and degradation (71–81). Indeed, we found that *N. castellii* siRNAs are susceptible to periodate oxidation and beta-elimination, thereby confirming that they are not methylated (Supplementary Figure S6A).

*Naumovozyma castellii* does have orthologs of several other factors thought to participate in RNAi in other species, including the QIP exonuclease, which removes the cleaved passenger strand in *Neurospora crassa* (27) and the autoantigen La RNA-binding protein, which is reported to enhance the multiple-turnover of RISC in Drosophila RNAi (82). However, disruption of the orthologs of the genes encoding QIP (*GFD2* and its paralog, *NCAS0A00350*), or the ortholog of La (*LHP1*) in an *N. castellii* RNAi-reporter strain had little effect on *GFP* silencing (Supplementary Figure S6B). Taken together, our results suggest that Xrn1p helps degrade the cleaved passenger strand and enhances the multiple turnover of RISC in budding yeast, thereby obviating the need for other proteins to perform these functions.

The general eukaryotic mRNA decay factors *DCP2* and *SKI3* have also been implicated in small-RNA–mediated gene-silencing pathways. *DCP2*, which encodes the mRNA decapping enzyme, is involved in miRNA-mediated silencing but not RNAi in Drosophila (83). *SKI3*, which encodes a component of the Ski complex, enables the cytoplasmic exosome to degrade 5′ target-slicing fragments in Drosophila (31). Although disrupting *DCP2* and *SKI3* affected the abundances of siRNAs in the cell (Supplementary Figure S2A), we found that the deletions of these genes in an *N. castellii* RNAi-reporter strain had little-to-no effect on *GFP* silencing (Figure 1E). These results suggest that each protein is either not involved in budding-yeast RNAi or has effects on the pathway that are undetectable in the *GFP*-silencing assay.

Xrn1p satisfies the expected criteria of a cofactor participating in the budding-yeast RNAi pathway. It is present in all known eukaryotes, playing important roles in RNA metabolism critical for cellular fitness, which explains why it was not lost in *S. cerevisiae* and other species that have lost RNAi. Although not thought to be an essential component of RNAi in any species, Xrn1 has been shown to function in RNAi and related pathways in diverse species, and our observations in budding yeast expand this repertoire, both with respect to its evolutionary reach and with respect to its functions within a single species. Indeed, studies in other species have focused on the role of Xrn1 on a particular step of the pathway, and thus it will be interesting to learn the degree to which Xrn1 has multiple functions in RNA-silencing pathways of other eukaryotes or whether these other lineages have evolved cofactors that assume these more specialized roles in more elaborated pathways.

## DATA AVAILABILITY

The *N. castellii* whole-genome sequencing data are available at the NCBI Sequence Read Archive (SRA) database under accession number PRJNA601242. The small-RNA and RNA sequencing data are available at the NCBI Gene Expression Omnibus (GEO) under accession number GSE143548.

## Supplementary Material

gkaa468_Supplemental_Files

## Notes

Present address: David E. Weinberg, Freenome, South San Francisco, CA 94080, USA.

Present address: Ines A. Drinnenberg, Institut Curi-PSL Research University, CNRS, Sorbonne Université, UMR3664, F-75005, Paris, France.

## References

[1] Tomari Y., Zamore P.D. Perspective: machines for RNAi. Genes Dev. 2005; 19:517–529. 15741316 10.1101/gad.1284105

[2] Malone C.D., Hannon G.J. Small RNAs as guardians of the genome. Cell. 2009; 136:656–668. 19239887 10.1016/j.cell.2009.01.045 PMC2792755

[3] Carthew R.W., Sontheimer E.J. Origins and Mechanisms of miRNAs and siRNAs. Cell. 2009; 136:642–655. 19239886 10.1016/j.cell.2009.01.035 PMC2675692

[4] Ghildiyal M., Zamore P.D. Small silencing RNAs: an expanding universe. Nat. Rev. Genet. 2009; 10:94–108. 19148191 10.1038/nrg2504 PMC2724769

[5] Cerutti H., Casas-Mollano J.A. On the origin and functions of RNA-mediated silencing: from protists to man. Curr. Genet. 2006; 50:81–99. 16691418 10.1007/s00294-006-0078-x PMC2583075

[6] Shabalina S.A., Koonin E.V. Origins and evolution of eukaryotic RNA interference. Trends Ecol. Evol. 2008; 23:578–587. 18715673 10.1016/j.tree.2008.06.005 PMC2695246

[7] Drinnenberg I.A., Fink G.R., Bartel D.P. Compatibility with killer explains the rise of RNAi-deficient fungi. Science. 2011; 333:1592. 21921191 10.1126/science.1209575 PMC3790311

[8] Drinnenberg I.A., Weinberg D.E., Xie K.T., Mower J.P., Wolfe K.H., Fink G.R., Bartel D.P. RNAi in budding yeast. Science. 2009; 326:544–550. 19745116 10.1126/science.1176945 PMC3786161

[9] Baulcombe D. RNA silencing in plants. Nature. 2004; 431:356–363. 15372043 10.1038/nature02874

[10] Sijen T., Fleenor J., Simmer F., Thijssen K.L., Parrish S., Timmons L., Plasterk R.H., Fire A. On the role of RNA amplification in dsRNA-triggered gene silencing. Cell. 2001; 107:465–476. 11719187 10.1016/s0092-8674(01)00576-1

[11] Smardon A., Spoerke J.M., Stacey S.C., Klein M.E., Mackin N., Maine E.M. EGO-1 is related to RNA-directed RNA polymerase and functions in germ-line development and RNA interference in *C. elegans*. Curr. Biol. 2000; 10:169–178. 10704412 10.1016/s0960-9822(00)00323-7

[12] Volpe T.A., Kidner C., Hall I.M., Teng G., Grewal S.I., Martienssen R.A. Regulation of heterochromatic silencing and histone H3 lysine-9 methylation by RNAi. Science. 2002; 297:1833–1837. 12193640 10.1126/science.1074973

[13] Cogoni C., Macino G. Gene silencing in *Neurospora crassa* requires a protein homologous to RNA-dependent RNA polymerase. Nature. 1999; 399:166–169. 10335848 10.1038/20215

[14] Xie Z., Johansen L.K., Gustafson A.M., Kasschau K.D., Lellis A.D., Zilberman D., Jacobsen S.E., Carrington J.C. Genetic and functional diversification of small RNA pathways in plants. PLoS Biol. 2004; 2:E104. 15024409 10.1371/journal.pbio.0020104 PMC350667

[15] Dalmay T., Hamilton A., Rudd S., Angell S., Baulcombe D.C. An RNA-dependent RNA polymerase gene in Arabidopsis is required for posttranscriptional gene silencing mediated by a transgene but not by a virus. Cell. 2000; 101:543–553. 10850496 10.1016/s0092-8674(00)80864-8

[16] Cerutti H., Casas-Mollano J.A. On the origin and functions of RNA-mediated silencing: from protists to man. Curr. Genet. 2006; 50:81–99. 16691418 10.1007/s00294-006-0078-x PMC2583075

[17] Liu Q., Rand T.A., Kalidas S., Du F., Kim H.E., Smith D.P., Wang X. R2D2, a bridge between the initiation and effector steps of the Drosophila RNAi pathway. Science. 2003; 301:1921–1925. 14512631 10.1126/science.1088710

[18] Liu Q., Paroo Z. Biochemical principles of small RNA pathways. Annu. Rev. Biochem. 2010; 79:295–319. 20205586 10.1146/annurev.biochem.052208.151733

[19] Iwasaki S., Kobayashi M., Yoda M., Sakaguchi Y., Katsuma S., Suzuki T., Tomari Y. Hsc70/Hsp90 chaperone machinery mediates ATP-dependent RISC loading of small RNA duplexes. Mol. Cell. 2010; 39:292–299. 20605501 10.1016/j.molcel.2010.05.015

[20] Miyoshi T., Takeuchi A., Siomi H., Siomi M.C. A direct role for Hsp90 in pre-RISC formation in Drosophila. Nat. Struct. Mol. Biol. 2010; 17:1024–1026. 20639883 10.1038/nsmb.1875

[21] Iwasaki S., Sasaki H.M., Sakaguchi Y., Suzuki T., Tadakuma H., Tomari Y. Defining fundamental steps in the assembly of the Drosophila RNAi enzyme complex. Nature. 2015; 521:533–536. 25822791 10.1038/nature14254

[22] Tomari Y., Matranga C., Haley B., Martinez N., Zamore P.D. A protein sensor for siRNA asymmetry. Science. 2004; 306:1377–1380. 15550672 10.1126/science.1102755

[23] Tsuboyama K., Tadakuma H., Tomari Y. Conformational activation of argonaute by distinct yet coordinated actions of the Hsp70 and Hsp90 chaperone systems. Mol. Cell. 2018; 70:722–729. 29775584 10.1016/j.molcel.2018.04.010

[24] Iki T., Yoshikawa M., Nishikiori M., Jaudal M.C., Matsumoto-Yokoyama E., Mitsuhara I., Meshi T., Ishikawa M. In vitro assembly of plant RNA-induced silencing complexes facilitated by molecular chaperone HSP90. Mol. Cell. 2010; 39:282–291. 20605502 10.1016/j.molcel.2010.05.014

[25] Gregory R.I., Chendrimada T.P., Cooch N., Shiekhattar R. Human RISC couples microRNA biogenesis and posttranscriptional gene silencing. Cell. 2005; 123:631–640. 16271387 10.1016/j.cell.2005.10.022

[26] Chendrimada T.P., Gregory R.I., Kumaraswamy E., Norman J., Cooch N., Nishikura K., Shiekhattar R. TRBP recruits the Dicer complex to Ago2 for microRNA processing and gene silencing. Nature. 2005; 436:740–744. 15973356 10.1038/nature03868 PMC2944926

[27] Maiti M., Lee H.C., Liu Y. QIP, a putative exonuclease, interacts with the Neurospora Argonaute protein and facilitates conversion of duplex siRNA into single strands. Genes Dev. 2007; 21:590–600. 17311884 10.1101/gad.1497607 PMC1820900

[28] Liu Y., Ye X., Jiang F., Liang C., Chen D., Peng J., Kinch L.N., Grishin N.V., Liu Q. C3PO, an endoribonuclease that promotes RNAi by facilitating RISC activation. Science. 2009; 325:750–753. 19661431 10.1126/science.1176325 PMC2855623

[29] Chang J.H., Xiang S., Xiang K., Manley J.L., Tong L. Structural and biochemical studies of the 5′→3′ exoribonuclease Xrn1. Nat. Struct. Mol. Biol. 2011; 18:270–276. 21297639 10.1038/nsmb.1984 PMC3075561

[30] Souret F.F., Kastenmayer J.P., Green P.J. AtXRN4 degrades mRNA in Arabidopsis and its substrates include selected miRNA targets. Mol. Cell. 2004; 15:173–183. 15260969 10.1016/j.molcel.2004.06.006

[31] Orban T.I., Izaurralde E. Decay of mRNAs targeted by RISC requires XRN1, the Ski complex, and the exosome. RNA. 2005; 11:459–469. 15703439 10.1261/rna.7231505 PMC1370735

[32] Lima W.F., De Hoyos C.L., Liang X.H., Crooke S.T. RNA cleavage products generated by antisense oligonucleotides and siRNAs are processed by the RNA surveillance machinery. Nucleic Acids Res. 2016; 44:3351–3363. 26843429 10.1093/nar/gkw065 PMC4838368

[33] Newbury S. The 5′-3′ exoribonuclease xrn-1 is essential for ventral epithelial enclosure during *C. elegans* embryogenesis. RNA. 2004; 10:59–65. 14681585 10.1261/rna.2195504 PMC1370518

[34] Chatterjee S., Fasler M., Büssing I., Grosshans H. Target-mediated protection of endogenous microRNAs in *C. elegans*. Dev. Cell. 2011; 20:388–396. 21397849 10.1016/j.devcel.2011.02.008

[35] Bartel D.P. Metazoan microRNAs. Cell. 2018; 173:20–51. 29570994 10.1016/j.cell.2018.03.006 PMC6091663

[36] Errampalli D., Patton D., Castle L., Mickelson L., Hansen K., Schnall J., Feldmann K., Meinke D. Embryonic lethals and T-DNA insertional mutagenesis in Arabidopsis. Plant Cell. 1991; 3:149–157. 12324593 10.1105/tpc.3.2.149 PMC159987

[37] Castle L.A., Errampalli D., Atherton T.L., Franzmann L.H., Yoon E.S., Meinke D.W. Genetic and molecular characterization of embryonic mutants identified following seed transformation in Arabidopsis. Mol. Gen. Genet. 1993; 241:504–514. 8264525 10.1007/BF00279892

[38] Bohmert K., Camus I., Bellini C., Bouchez D., Caboche M., Benning C. *AGO1* defines a novel locus of Arabidopsis controlling leaf development. EMBO J. 1998; 17:170–180. 9427751 10.1093/emboj/17.1.170 PMC1170368

[39] Tabara H., Sarkissian M., Kelly W.G., Fleenor J., Grishok A., Timmons L., Fire A., Mello C.C. The *rde-1* gene, RNA interference, and transposon silencing in *C. elegans*. Cell. 1999; 99:123–132. 10535731 10.1016/s0092-8674(00)81644-x

[40] Vastenhouw N.L., Fischer S.E., Robert V.J., Thijssen K.L., Fraser A.G., Kamath R.S., Ahringer J., Plasterk R.H. A genome-wide screen identifies 27 genes involved in transposon silencing in *C. elegans*. Curr. Biol. 2003; 13:1311–1316. 12906791 10.1016/s0960-9822(03)00539-6

[41] Kennedy S., Wang D., Ruvkun G. A conserved siRNA-degrading RNase negatively regulates RNA interference in *C. elegans*. Nature. 2004; 427:645–649. 14961122 10.1038/nature02302

[42] Kim J.K., Gabel H.W., Kamath R.S., Tewari M., Pasquinelli A., Rual J.F., Kennedy S., Dybbs M., Bertin N., Kaplan J.M. et al. Functional genomic analysis of RNA interference in *C. elegans*. Science. 2005; 308:1164–1167. 15790806 10.1126/science.1109267

[43] Parry D.H., Xu J., Ruvkun G. A whole-genome RNAi Screen for *C. elegans* miRNA pathway genes. Curr. Biol. 2007; 17:2013–2022. 18023351 10.1016/j.cub.2007.10.058 PMC2211719

[44] Zhou R., Hotta I., Denli A.M., Hong P., Perrimon N., Hannon G.J. Comparative analysis of argonaute-dependent small RNA pathways in Drosophila. Mol. Cell. 2008; 32:592–599. 19026789 10.1016/j.molcel.2008.10.018 PMC2615197

[45] Smibert P., Bejarano F., Wang D., Garaulet D.L., Yang J.S., Martin R., Bortolamiol-Becet D., Robine N., Hiesinger P.R., Lai E.C. A Drosophila genetic screen yields allelic series of core microRNA biogenesis factors and reveals post-developmental roles for microRNAs. RNA. 2011; 17:1997–2010. 21947201 10.1261/rna.2983511 PMC3198593

[46] Pressman S., Reinke C.A., Wang X., Carthew R.W. A systematic genetic screen to dissect the MicroRNA pathway in Drosophila. G3. 2012; 2:437–448. 22540035 10.1534/g3.112.002030 PMC3337472

[47] Amberg D.C., Burke D.J., Strathern J.N. Ethyl Methane Sulfonate (EMS) mutagenesis. CSH Protoc. 2006; 2006:doi:10.1101/pdb.prot4180. 10.1101/pdb.prot4180 22485581

[48] Hoffman C.S., Winston F. A ten-minute DNA preparation from yeast efficiently releases autonomous plasmids for transformation of *Escherichia coli*. Gene. 1987; 57:267–272. 3319781 10.1016/0378-1119(87)90131-4

[49] Schneider C.A., Rasband W.S., Eliceiri K.W. NIH image to ImageJ: 25 years of image analysis. Nat. Methods. 2012; 9:671–675. 22930834 10.1038/nmeth.2089 PMC5554542

[50] Fang W., Bartel D.P. The menu of features that define primary microRNAs and enable de novo design of microRNA genes. Mol. Cell. 2015; 60:131–145. 26412306 10.1016/j.molcel.2015.08.015 PMC4613790

[51] Rio D.C. Filter-binding assay for analysis of RNA-protein interactions. Cold Spring Harb. Protoc. 2012; 2012:1078–1081. 23028069 10.1101/pdb.prot071449

[52] Wee L.M., Flores-Jasso C.F., Salomon W.E., Zamore P.D. Argonaute divides its RNA guide into domains with distinct functions and RNA-binding properties. Cell. 2012; 151:1055–1067. 23178124 10.1016/j.cell.2012.10.036 PMC3595543

[53] Chen G.R., Sive H., Bartel D.P. A seed mismatch enhances Argonaute2-Catalyzed cleavage and partially rescues severely impaired cleavage found in fish. Mol. Cell. 2017; 68:1095–1107. 29272705 10.1016/j.molcel.2017.11.032 PMC5821252

[54] Balciunas D., Ronne H. Three subunits of the RNA polymerase II mediator complex are involved in glucose repression. Nucleic Acids Res. 1995; 23:4421–4425. 7501465 10.1093/nar/23.21.4421 PMC307399

[55] Hengartner C.J., Thompson C.M., Zhang J., Chao D.M., Liao S.M., Koleske A.J., Okamura S., Young R.A. Association of an activator with an RNA polymerase II holoenzyme. Genes Dev. 1995; 9:897–910. 7774808 10.1101/gad.9.8.897

[56] Liao S.M., Zhang J., Jeffery D.A., Koleske A.J., Thompson C.M., Chao D.M., Viljoen M., van Vuuren H.J., Young R.A. A kinase-cyclin pair in the RNA polymerase II holoenzyme. Nature. 1995; 374:193–196. 7877695 10.1038/374193a0

[57] Kuchin S., Yeghiayan P., Carlson M. Cyclin-dependent protein kinase and cyclin homologs *SSN3* and *SSN8* contribute to transcriptional control in yeast. Proc. Natl. Acad. Sci. U.S.A. 1995; 92:4006–4010. 7732022 10.1073/pnas.92.9.4006 PMC42091

[58] Anderson J.S., Parker R.P. The 3′ to 5′ degradation of yeast mRNAs is a general mechanism for mRNA turnover that requires the SKI2 DEVH box protein and 3′ to 5′ exonucleases of the exosome complex. EMBO J. 1998; 17:1497–1506. 9482746 10.1093/emboj/17.5.1497 PMC1170497

[59] Dunckley T., Parker R. The DCP2 protein is required for mRNA decapping in *Saccharomyces cerevisiae* and contains a functional MutT motif. EMBO J. 1999; 18:5411–5422. 10508173 10.1093/emboj/18.19.5411 PMC1171610

[60] Teixeira D., Parker R. Analysis of P-body assembly in *Saccharomyces cerevisiae*. Mol. Biol. Cell. 2007; 18:2274–2287. 17429074 10.1091/mbc.E07-03-0199 PMC1877105

[61] Brönner C., Salvi L., Zocco M., Ugolini I., Halic M. Accumulation of RNA on chromatin disrupts heterochromatic silencing. Genome Res. 2017; 27:1174–1183. 28404620 10.1101/gr.216986.116 PMC5495069

[62] Szachnowski U., Andus S., Foretek D., Morillon A., Wery M. Endogenous RNAi pathway evolutionarily shapes the destiny of the antisense lncRNAs transcriptome. Life Sci. Alliance. 2019; 2:e201900407. 31462400 10.26508/lsa.201900407 PMC6713810

[63] Flores-Jasso C.F., Salomon W.E., Zamore P.D. Rapid and specific purification of Argonaute-small RNA complexes from crude cell lysates. RNA. 2013; 19:271–279. 23249751 10.1261/rna.036921.112 PMC3543083

[64] Haley B., Zamore P.D. Kinetic analysis of the RNAi enzyme complex. Nat. Struct. Mol. Biol. 2004; 11:599–606. 15170178 10.1038/nsmb780

[65] Salomon W.E., Jolly S.M., Moore M.J., Zamore P.D., Serebrov V. Single-molecule imaging reveals that argonaute reshapes the binding properties of its nucleic acid guides. Cell. 2015; 162:84–95. 26140592 10.1016/j.cell.2015.06.029 PMC4503223

[66] Nakanishi K., Weinberg D.E., Bartel D.P., Patel D.J. Structure of yeast Argonaute with guide RNA. Nature. 2012; 486:368–374. 22722195 10.1038/nature11211 PMC3853139

[67] Weinberg D.E., Nakanishi K., Patel D.J., Bartel D.P. The inside-out mechanism of Dicers from budding yeasts. Cell. 2011; 146:262–276. 21784247 10.1016/j.cell.2011.06.021 PMC3169304

[68] MacRae I.J., Zhou K., Doudna J.A. Structural determinants of RNA recognition and cleavage by Dicer. Nat. Struct. Mol. Biol. 2007; 14:934–940. 17873886 10.1038/nsmb1293

[69] Zhang H., Kolb F.A., Jaskiewicz L., Westhof E., Filipowicz W. Single processing center models for human Dicer and bacterial RNase III. Cell. 2004; 118:57–68. 15242644 10.1016/j.cell.2004.06.017

[70] Macrae I.J., Zhou K., Li F., Repic A., Brooks A.N., Cande W.Z., Adams P.D., Doudna J.A. Structural basis for double-stranded RNA processing by Dicer. Science. 2006; 311:195–198. 16410517 10.1126/science.1121638

[71] Yu B., Yang Z., Li J., Minakhina S., Yang M., Padgett R.W., Steward R., Chen X. Methylation as a crucial step in plant microRNA biogenesis. Science. 2005; 307:932–935. 15705854 10.1126/science.1107130 PMC5137370

[72] Yang Z., Ebright Y.W., Yu B., Chen X. HEN1 recognizes 21–24 nt small RNA duplexes and deposits a methyl group onto the 2′ OH of the 3′ terminal nucleotide. Nucleic Acids Res. 2006; 34:667–675. 16449203 10.1093/nar/gkj474 PMC1356533

[73] Li J., Yang Z., Yu B., Liu J., Chen X. Methylation protects miRNAs and siRNAs from a 3′-end uridylation activity in Arabidopsis. Curr. Biol. 2005; 15:1501–1507. 16111943 10.1016/j.cub.2005.07.029 PMC5127709

[74] Vagin V.V., Sigova A., Li C., Seitz H., Gvozdev V., Zamore P.D. A distinct small RNA pathway silences selfish genetic elements in the germline. Science. 2006; 313:320–324. 16809489 10.1126/science.1129333

[75] Horwich M.D., Li C., Matranga C., Vagin V., Farley G., Wang P., Zamore P.D. The Drosophila RNA methyltransferase, DmHen1, modifies germline piRNAs and single-stranded siRNAs in RISC. Curr. Biol. 2007; 17:1265–1272. 17604629 10.1016/j.cub.2007.06.030

[76] Houwing S., Kamminga L.M., Berezikov E., Cronembold D., Girard A., van den Elst H., Filippov D.V., Blaser H., Raz E., Moens C.B et al. A role for Piwi and piRNAs in germ cell maintenance and transposon silencing in Zebrafish. Cell. 2007; 129:69–82. 17418787 10.1016/j.cell.2007.03.026

[77] Kamminga L.M., Luteijn M.J., den Broeder M.J., Redl S., Kaaij L.J., Roovers E.F., Ladurner P., Berezikov E., Ketting R.F. Hen1 is required for oocyte development and piRNA stability in zebrafish. EMBO J. 2010; 29:3688–3700. 20859253 10.1038/emboj.2010.233 PMC2982757

[78] Kirino Y., Mourelatos Z. The mouse homolog of HEN1 is a potential methylase for Piwi-interacting RNAs. RNA. 2007; 13:1397–1401. 17652135 10.1261/rna.659307 PMC1950760

[79] Kirino Y., Mourelatos Z. Mouse Piwi-interacting RNAs are 2′-O-methylated at their 3′ termini. Nat. Struct. Mol. Biol. 2007; 14:347–348. 17384647 10.1038/nsmb1218

[80] Saito K., Sakaguchi Y., Suzuki T., Suzuki T., Siomi H., Siomi M.C. Pimet, the Drosophila homolog of HEN1, mediates 2′-O-methylation of Piwi- interacting RNAs at their 3′ ends. Genes Dev. 2007; 21:1603–1608. 17606638 10.1101/gad.1563607 PMC1899469

[81] Ji L., Chen X. Regulation of small RNA stability: methylation and beyond. Cell Res. 2012; 22:624–636. 22410795 10.1038/cr.2012.36 PMC3317568

[82] Liu Y., Tan H., Tian H., Liang C., Chen S., Liu Q. Autoantigen La promotes efficient RNAi, antiviral response, and transposon silencing by facilitating multiple-turnover RISC catalysis. Mol. Cell. 2011; 44:502–508. 22055194 10.1016/j.molcel.2011.09.011 PMC3229097

[83] Rehwinkel J., Behm-Ansmant I., Gatfield D., Izaurralde E. A crucial role for GW182 and the DCP1:DCP2 decapping complex in miRNA-mediated gene silencing. RNA. 2005; 11:1640–1647. 16177138 10.1261/rna.2191905 PMC1370850

