# Supplementary material for: Xrn1p acts at multiple steps in the budding-yeast RNAi pathway to enhance the efficiency of silencing
Source: Nucleic Acids Res. 2020 Jun 5;48(13):7404–20. doi: 10.1093/nar/gkaa468 (PMC7528652; doi:10.1093/nar/gkaa468)
Supplement: gkaa468_Supplemental_Files [file gkaa468_supplemental_files.zip › Xrn1 in RNAi - Supplementary Material - NAR - Resubmission - Final.pdf]

SUPPLEMENTARY MATERIALS AND METHODS

Plasmid construction

Integrating plasmids for N. castellii RNAi selection strains

pIp-strongSC_GFP, the integrating plasmid containing the *GFP*-silencing construct, was constructed previously (1). To construct pRS403-ScerURA3hp-HIS3-PEST, the integrating plasmid containing the *URA3*-silencing construct, the *N. castellii* genomic sequence downstream of the ORF NCAS0C02950 was amplified (primers: Ncas_Int679_For 5′ GGCAAATTTGTATGAGGGATAAA and Ncas_Int679_Rev 5′ TAATTCGATTACGTTAGCTGTT) and cloned into pRS403-pGAL1-hpSC_URA3 (1) using PsiI and NaeI restriction enzymes (New England Biolabs, NEB). In addition, the *PEST* sequence from *S. cerevisiae CLN2* was appended to the C-terminal codon of the *HIS3* gene using the In-Fusion Cloning Kit (Clontech) as previously described (2), which increased the stringency of the selection. To construct pRS405-ScerHIS3hp, the integrating plasmid containing the *HIS3*-silencing construct, a 286 bp of *HIS3* sequence from pRS403-pGAL1-hpSC_URA3 was amplified (primers: HIS3_hp_For 5′ AAAAGCTTGACCGAGAGCAA and HIS3_hp_Rev 5′ GCGTATTACAAATGAAACCAAGATTCA) and initially cloned into pRS405 (3) between HindIII and XhoI (NEB). A DNA segment containing the *GAL1* promoter, the *HIS3* sequence in the antisense direction, and the *Schizosaccharomyces pombe rad9* intron was synthesized via fusion PCR (4) and inserted between BamHI and PstI (NEB). The *N. castellii* genomic sequence in between the ORFs NCAS0D00680 and NCAS0D00690 was amplified (primers: Ncas_Int618_For 5′-GTTC**GCCGGC**CTTCCCGCGCTATGAAATTA and Ncas_Int618_Rev 5′-ATCA**GGCGCC**GAGCATAACCGCTCAAATGC) and inserted between the NaeI and KasI

(NEB) restriction sites. To construct pRS404-NcasAGO1, the integrating plasmid containing *N. castellii AGO1* (NCAS0J02110), *N. castellii* genomic sequence downstream of the ORF NCAS0C00690 was amplified (primers: Ncas_Int696_For: 5′-GGCC**GGTACC**AATTCATCTAGCAGGATGTAAAATG; Ncas_Int696_Rev: 5′-GAAA**GCCGGC**GTAGAGCATGCGAGGTTTGG) and inserted between the KpnI and NaeI (NEB) restriction sites in pRS404 (3). The *AGO1* ORF along with its upstream and downstream sequence was amplified (primers: AGO1-Intergenic-For 5′-GCTG**GAGCTC**TGAACGTGTGGAAGACCAAA; AGO1-Intergenic-Rev 5′-ATGA**CTCGAG**AGTGGCTAACGGCAACATATC) and inserted between the SacI and XhoI (NEB) restriction sites of pRS404. To construct pRS402-NcasDCR1, the integrating plasmid containing *N. castelii DCR1* (NCAS0C00230), *N. castellii* genomic sequence upstream of the ORF NCAS0E03540 was amplified (primers: Ncas_Int701_For 5′-ATTC**GGATCC**TGCAGGCTGTTTGCTGTACT; Ncas_Int701_Rev 5′-GGTG**GCGGCCGC**GGGGTAACTATCCGCGTCTAA) and inserted between the BamHI and NotI (NEB) restriction sites in pRS402 (5). The *DCR1* ORF along with its upstream and downstream sequence was amplified (primers: DCR1-Intergenic-For 5′-CCCC**CTCGAG**TTTGTAAAGAAATTGATGCTTCG; DCR1-Intergenic-Rev 5′-TGCA**GGATCC**GAATCTGGTATGGGATCATATTGG) and inserted between the XhoI and BamHI (NEB) restriction sites of pRS402.

Plasmids for protein expression strains

pYES2.1-FLAG3-SUMO-TEV-NcasAGO1, which expresses N-terminally FLAG- and SUMO-tagged *AGO1*, was constructed by creating a gBlock gene fragment (IDT) of 3x-FLAG, SUMO,

and TEV sequences and cloning it immediately downstream of the *GAL1* promoter in pYES2.1 by Gibson Assembly (NEB). The *AGO1* CDS lacking the start codon was then inserted immediately downstream of the 3x-FLAG-SUMO-TEV construct by Gibson Assembly. pYES2.1-NcasXRN1-TEV-SUMO-FLAG3, which expresses C-terminally FLAG- and SUMO-tagged *XRN1* was constructed in the same manner, except the full *XRN1* CDS was inserted in between the *GAL1* promoter and the TEV-SUMO-3x-FLAG sequence. QuikChange Site-Directed Mutagenesis (Agilent) was used to generate pYES2.1-NcasXRN1(D206N-D208N)-TEV-SUMO-FLAG3, for which active-site aspartates at amino acid positions 206 and 208 of *XRN1* were substituted with asparagine (QuikChange primers, with changed nucleotides bolded: sense 5′-GAAGAATAACAGCAGAGAAGGATTTTGATGAAAATACTAGACACTGTATTTATGGATTA**A**TGCA**A**TCTAATAATATTGGGGCTTTCCACTCATGCTCCACATTTCGCACTGTTAAGAG; antisense, reverse complement of this sequence).

Strain construction

N. castellii RNAi selection strains, RNAi mutant strains and derivatives

The parental *N. castellii* genetic selection strains DPB537 (*MAT* **a**) and DPB507 (*MAT* α) were derived from the original progenitor strains DPB004 and DPB005 (1), respectively. *ADE2* (NCAS0H02150), *HIS3* (NCAS0B01740), *LEU2* (NCAS0C04870) in both strains, as well as *LYS2* (NCAS0I01040) in DPB537 and *MET17* (NCAS0A05810) in DPB507, were disrupted with loxP-kanMX6-loxP modules of plasmid pUG6 (6) that had been fused via PCR (7) with ~400–500-bp targeting arms on both sides of the cassette followed by expression of Cre recombinase, as described (1). *TRP1* (NCAS0E01400) was disrupted using the hygromycin

cassette of pAG32 (8), as described (1). The GFP(S65T)-kanMX6 module was inserted into the genome as described (1). The *GFP* silencing construct (pIp-strongSC_GFP) was integrated upstream of the *ADH2* ORF (NCAS0I02350), as described (1) in these strains and all strains containing this *GFP* silencing construct. The integrating plasmids generated from the pRS402, pRS403, pRS404, and pRS405 backbones were each digested with restriction enzymes that made single cuts in their regions of homology with the *N. castellii* genome and then transformed into *N. castellii* independently. Transformants were selected on appropriate synthetic media based on the selectable markers they possessed. Strain DPB325 was derived from strain DPB313, which was previously constructed (1), by disrupting *ADE1* (NCAS0I01850) with a cassette consisting of ~400–500-bp targeting arms upstream and downstream of the *ADE1* ORF amplified from genomic DNA attached to the kanMX cassette of pFA6a via fusion PCR. Strain DPB079 was derived from strain DPB005 using the loxP-kanMX6-loxP modules discussed above to disrupt *HIS3* and *MET17*. DPB534 was created by amplifying regions upstream and downstream of *DCR1* (primers: Upstream arm: 5′-TAATTAGCGGGCCATTATACCG; 5′-TTGTATAATTGCGTGTAGGTCACTT; Downstream arm: 5′-AAATGATATTTATGCACCTTTT; 5′-TTGAAGTGGAAGCCAGAATG) and appending them to a *NAT* cassette from pAG25 (8) via fusion PCR. The *XRN1* (NCAS0C04170) disruption cassette was created by amplifying genomic regions flanking the *XRN1* ORF (primers: Upstream arm: 5′-TTGCTCTCGAGCTTAGCAAC; 5′-TGGAATACCCATGATTAAAATGAA; Downstream arm: 5′-ATGCCACCACCACATCCATA; 5′-AGATGGGATGGCGTTGATAG) and adding them to a *NAT* cassette via fusion PCR as described above. Strains DPB510, DPB535, and DPB541 were created by transforming the *XRN1-NAT* disruption cassette into strains DPB507, DPB079, and DPB537, respectively, and transformed and selected on YPD +

NAT solid media. Similarly, the *SRB8* (NCAS0D04810) disruption cassettes were created by amplifying flanking genomic regions (Upstream arm: 5′-AGGGGAAGAATGTTTTATTTGTTT; 5′-AAAAAGTTGGGGGTTCTCTAGT; Downstream arm: 5′-GCACGACGATTTTGAGACAA; 5′-CCATCTTCAAAGAGGCCCCATA) and adding them to a *NAT* cassette or a *N. castellii LYS2* cassette consisting of the ORF with upstream and downstream regions (5′-ACCAATCGACCTTTGGATTA; 5′-TGATTCGGTTTTACAAATTGTTC) by fusion PCR. Strains DPB578 and DPB582 were created by transforming the *SRB8-NAT* or *SRB8-LYS2* followed by *XRN1-NAT* disruption cassettes, respectively, into strain DPB537 and then selected as discussed above.

RNA decay mutants

The RNA decay mutant strains DPB334, DPB336, DPB337 and DPB339 were generated from the parental strains DPB331 and DPB333 (1). *AGO1* was disrupted in strain DPB442 with a hygromycin cassette as previously described (1). Flanking regions of *DCR1* (primers: Upstream arm: 5′-ACTGATCCGGAAAAGAGAGT; 5′-TCACTTGATCTGTTGCTGGAGG; Downstream arm: 5′-AGGCATTGCAACAATCTGTGA; 5′-GAGTTTATCGATGATACCATTGAAGG), *XRN1* (primers: Upstream arm: 5′-TTGCTCTCGAGCTTAGCAAC; 5′-TGGAATACCCATGATTAAAATGAA; Downstream arm: 5′-ATGCCAATGCCACCACCACA; 5′-AGATGGGATGGCGTTGATAG), *SKI3* (NCAS0A15300 – primers: Upstream arm: 5′-TTGCACTGGTTGACTCCTCT; 5′-CAATTGCTTCACTTCCGACA; Downstream arm: 5′-GGCTGTTATGGCTTTGAAGG; 5′-TCAAAATAGGCTTATCCGACGA) and *DCP2* (NCAS0G02500 – primers: Upstream arm: 5′-ATATGGCAGGCGTGTTTGGT; 5′-AGGGTGTGACAGAGGAAAAGT; Downstream arm: 5′-

GGTTACGATCTATTGCTGTCATTC; 5′-CCATCAAACTCACACATTCAAAA) were amplified from genomic DNA. These flanking arms were appended to the hygromycin cassette of plasmid pAG32 via fusion PCR. These disruption cassettes were transformed into strains DPB331 and DPB333 and transformants were selected for on YPD + hygromycin solid media.

Deletions of orthologs of factors that act in RNAi of other species

Flanking regions of *GFD2* (NCAS0B08880 – primers: Upstream arm: 5′-TGCTTCTTCTACAATGGTCAAGT; 5′-CTGCTAATGGTATGCTTATTGCCTTC; Downstream arm: 5′-TGTTAGATAGATAAAATTTCATTAACGTAT; 5′-GCAATTGATCAACGAGAAGGA) were amplified from genomic DNA. These flanking arms were appended to the hygromycin cassette of plasmid pAG32 via fusion PCR. Flanking regions of *NCAS0A00350* (primers: Upstream arm: 5′-CTGTCCCCATCGCAGGTAT; 5′-ACAAGATGAGTGATTGATGTATGTG; Downstream arm: 5′-AAGTAACATTAATAATATTCTTTAAAATGAAC; 5′-CTACGTCCAAAAACTTATCAAGGA) were amplified from genomic DNA. These flanking arms were appended to the *HIS3* cassette via fusion PCR. The *NCAS0A00350-HIS3* disruption cassette was transformed into strain DPB056 and transformants were selected for on SD – His and then the *GFD2-HYG* disruption cassette was transformed into these selected intermediate strains and transformants were selected for on YPD + hygromycin solid media to create strain DPB504. Strain DPB532 was created by amplifying regions upstream and downstream of *LHP1* (NCAS0A02380 – primers: Upstream arm: 5′-CAGAGAGAATGAAGTAAAGTAAGTTGG; 5′-TGAGTAAGGGTGGTTACCGT; Downstream arm: 5′-AGTTATCACTGAATGAAGAATTTTA; 5′-GTCGTTGGGGTCATTGATTCA) and

appending them to a *NAT* cassette from pAG25 (8) via fusion PCR. The *LHP1-NAT* disruption cassette was transformed into strain DPB333 and transformants were selected on YPD + NAT solid media.

Tagged Ago1p, Dcp2p, and Xrn1p strains

Diploid strain DPB202 was made by transforming the previously synthesized *AGO1* hygromycin disruption cassette into the diploid strain Y235 (1) and selecting for transformants on YPD + hygromycin solid media. An N-terminal-kanMX-pGAL1-3x-HA-AGO1 tagging construct was generated by amplifying a region of the pFA6a-kanMX6-pGAL1-3HA plasmid (9) and joining it via fusion PCR to ~400–500-bp targeting arms corresponding to the regions immediately upstream of the *AGO1* CDS and immediately downstream of the *AGO1* start codon. To create diploid strain DPB201, this *AGO1* tagging construct was transformed into diploid strain DPB202 and transformants were selected for on YPD + G418 solid media. In strains DPB215 and DPB221, *XRN1* was C-terminally tagged with 3x-HA and kanMX was created via fusion PCR (7). Genomically encoded, N-terminally tagged *eGFP(S65T)-AGO1* was created by replacing the start codon of *AGO1* with *S. cerevisiae URA3* expression cassette amplified from pYES2.1 (Invitrogen) (1) and appended to the *AGO1* arms of homology described above. These cassettes were transformed into strain DPB211 and transformants were selected on SD – Ura. The eGFP(S65T) construct was amplified from strain DPB314 (1) and transformed into the intermediate strain containing the *URA3* cassette in place of the *AGO1* start codon and transformants were selected on media containing 5-FOA. In strains DPB231 and DPB234, *XRN1* and *DCP2* were C-terminally tagged with cassettes containing mCherry and kanMX for selection from pBS34 (10) with fusion PCR.

Strains for small-RNA and RNA sequencing

DPB220 served as the parental strain for all strains used for small-RNA and RNA sequencing. To create DPB228, the *AGO1* slicing-mutant strain, the sequence encoding the PIWI domain of *AGO1* (NCAS0J02110 - Ncas_Chr10: 404375–405853) was replaced by transforming with *S. cerevisiae URA3* expression cassette amplified from pYES2.1 flanked by ~400–500-bp arms of homology, as described before. QuikChange Site-Directed Mutagenesis (Agilent) was used to change *AGO1* coding sequence in pYES2.1-*AGO1* (1) to encode arginine in place of aspartate at amino acid position 1247 (D1247R) (QuikChange primers, changed codon is in bold: 5′-GTCGCATCTCCTGTTTATTACGCT**CGT**TTATTGTGTGAACGTGGTGCTGCA; 5′-TGCAGCACCACGTTCACACAATAA**ACG**AGCGTAATAAACAGGAGATGCGAC). The *ago1* D1247R construct was amplified and transformed into the intermediate strain, and transformants were selected as described above. To create DPB622, the Δ*xrn1* strain, the flanking regions of *XRN1* previously used for the hygromycin disruption cassette were amplified from genomic DNA and appended to the kanMX cassette of pFA6a via fusion PCR. This disruption cassette was transformed into strain DPB220 and transformants were selected on YPD + G418.

Protein-expression strains

The *S. cerevisiae* protein-expression strains DPB597, DPB598, DPB1100, and DPB1101 were synthesized using the parental yRH101 strain (a gift from Stephen Bell, MIT), which was derived from ySC7 (11). To create strain DPB597, *XRN1* was deleted in yRH101 using a CRISPR–Cas9 system adapted for use in *S. cerevisiae* (12,13). The template for the sgRNA

targeting *XRN1* (Genome matching sequence in bold. 5′-GATCG**AAAAGGTTTTAGGTTACGCGG**; 5′-**AAAACCGCGTAACCTAAAACCTTTT**C) was cloned into plasmid pV1382 and transformed into yRH101 along with a repair template made from hybridizing oligos with homology upstream and downstream of the *XRN1* ORF (5′-AAAAATCAACACTTGTAACAACAGCAGCAACAAATATATATCAGTACGGTAACATACGAC; 5′-GATATACTATTAAAGTAACCTCGAATATACTTCGTTTTAGTCGTATGTTACCGTACTGA). To create strains DPB598, DPB1100, and DPB1101, plasmids pYES2.1-FLAG3-SUMO-TEV-NcasAGO1, pYES2.1-NcasXRN1-TEV-SUMO-FLAG3, and pYES2.1-NcasXRN1(D206N-D208N)-TEV-SUMO-FLAG3, respectively, were transformed into strain DPB597, and transformants were selected on SD – Ura.

Flow cytometry of strains with RNA-decay mutations

Non-inducing SC (+ 2% glucose) 3 mL cultures were inoculated with strains that contained neither *GFP* nor a *GFP*-silencing construct (DPB211, DPB443, DPB442, DPB620, DPB601, DPB608), or contained *GFP* and an empty *GFP*-silencing construct (DPB331, DPB334, DPB337, DPB625, DPB469, DPB611), and 3 mL inducing SC (+ 2% galactose) cultures of strains that contained *GFP* and a *GFP*-silencing construct (DPB333, DPB336, DPB339, DPB629, DPB471, DPB613) and incubated at 25°C until they reached saturation. Strains with *DCP2* disrupted were inoculated before the other cultures, as they took > 24 h to reach saturation. Saturated cultures were diluted to OD_{600} 0.04 in 13 mL inducing or non-inducing media and grown until all cultures were in mid-log phase (OD_{600} 0.5 – 0.8). Flow cytometry was

then performed on 500 µL of each culture as described for other strains. Strains used in Figure 1E were DPB211, DPB443, DPB442, DPB620, DPB331, DPB334, DPB337, DPB625, DPB333, DPB336, DPB339, DPB629, DPB601, DPB608, DPB469, DPB611, DPB471, DPB613. Strains used in Supplemental Figure S6B were DPB075, DPB077, DPB056, DPB504 for Δ*gfd2* Δ*NCAS0A00350*; and DPB005, DPB331, DPB333, and DPB532 for *lhp1*.

Serial-dilution spot assay

Inducing SC (+ 2% galactose) cultures of *N. castellii* strains were inoculated and incubated at 30°C for 12 h. These cultures were centrifuged for 5 min at 3000 x *g*, resuspended in SC (+ 2% galactose), and used to re-inoculate new cultures at OD_{600} 0.2. Cultures were grown until OD_{600} 1.0, centrifuged for 5 min at 3000 x g, and resuspended in water. Cells were diluted to 2 x 10^6 colony forming units (CFU)/mL and subsequent tenfold dilutions were performed. Cells (5 µL) were plated on SC (+ 2% galactose) and SGal + Lys agar plates and grown for at least ten days. Strains from top to bottom in Figure 1C are DPB537, DPB541, DPB578, DPB582.

Genome sequencing and analysis

The *N. castellii* genome was downloaded from Yeast Genome Order Browser (YGOB - http://ygob.ucd.ie/). Sequences of the integrating plasmids containing *GFP*, *URA3* and *HIS3-PEST* along with their silencing constructs, as well as the integrating plasmids containing extra copies of *AGO1* and *DCR1* were inserted into the FASTA file. STAR (v.2.4) was used to create an indexed genome (14). Reads were aligned to the genome using STAR (v.2.4) with the parameters '--outFilterMismatchNmax 4 --clip3pAdapterSeq CTGTCTCTTATAC --readMatesLengthsIn NotEqual --alignIntronMax 1 --alignMatesGapMax 1000'. Samtools (v1.3)

‘view’, ‘sort’, and ‘rmdup’ commands were used to sort the BAM file and remove duplicate reads (15). Samtools ‘mpileup’ command with the parameters ‘-t DP -t SP -ug’ was used to make a BCF file. Bcftools (v1.3) ‘call’ command with the parameters ‘-vm’ was used to call variants for each strain. A custom Python script was employed to remove the parental strain variants from each of the mutant strain variants, and to identify and select the variants that were called as homozygous by bcftools (as these were haploid strains). Custom Python scripts were used to characterize each of the remaining variants as being either genic (present in an ORF) or intergenic, and as being either exonic or intronic. Exonic ORF variants were further characterized as being either synonymous, nonsynonymous, or frameshift mutations, and the nonsynonymous mutations were classified as being either missense or nonsense mutations using custom Python scripts. The variants and the mutated genes in each of the mutant strains were compiled. Variants that occurred in more than two mutant strains were removed. All frameshift variants that occurred in repetitive tracts of DNA for which the variant caller (bcftools) identified more than one position of the repeat as being mutated were also removed. The remaining variants were considered bona fide mutations that occurred during either mutagenesis or selection of the mutant strains.

Ago1p over-expression, immunoprecipitation and mass spectrometry

Overnight YPD cultures of diploid strains DPB201 and DPB202 were inoculated, grown to saturation, diluted to OD_{600} 0.05 in 250 mL YPD and grown until OD_{600} 1.0. Cultures were then induced by adding galactose to 2% and grown at 30°C for 20 h before harvesting to make 400–500 mg pellets. An equal volume of lysis buffer (50 mM HEPES pH 7.6, 300 mM NaCl, 0.1 mM EDTA, 0.1 mM EGTA, 0.25% NP-40) was added to the pellets. Cells were lysed with acid-

washed glass-bead beatings of 4 x 45 seconds with >4 min between bead beatings. 100 μL 50% anti-HA affinity matrix (Roche 11815016001) was added to the lysate and incubated 12 h at 4°C in experiment 1 and 60 μL of 50% anti-HA-agarose (Sigma A2095) was added and incubated for 2 h at 4°C in experiment 2. The affinity agarose was then washed four times with lysis buffer and all liquid was removed after the last wash with a needle. Proteins were eluted with 1x volume of HA peptide (1 μg/μL) with incubation at 37°C for 15 min in experiment 1 and with incubation at 4°C for 12 h in experiment 2. Liquid was removed from the beads, 2x SDS buffer was added and then boiled for 5 min and samples were run on a 4-12% Tris-Glycine gel.

A silver stain of the Tris-Glycine gel was performed using Pierce Silver Stain for Mass Spectrometry (ThermoFisher Scientific 24600). Each sample band in the gel was cut into ~5 mm squares and was washed overnight in 50% methanol. These gel fragments were subsequently washed with a solution of 47.5% methanol, 5% acetic acid for 2 h and then dehydrated with acetonitrile and dried in a speed-vac. To reduce disulfide bonds, the dried gel pieces were incubated in 30 μL 100 mM ammonium bicarbonate with 10 mM dithiothreitol for 30 min and were then incubated in 30 μL of 100 mM ammonium bicarbonate and 100 mM iodoacetmide for cysteine alkylation. The gel fragments were then washed with acetonitrile, 100 mM ammonium bicarbonate and acetonitrile, in sequence, and dried in a speed-vac. The fragments were then rehydrated with a sufficient volume of 50 mM ammonium bicarbonate. To digest the proteins, trypsin was added (final concentration of 20 ng/μL), incubated on ice for 10 min, and then digested overnight at 37°C with gentle shaking. The digested peptides were extracted from the gel slices by sequential 10 min incubations at 37°C with shaking of the slices with 50 μL of 50 mM ammonium bicarbonate, then 50 μL of a solution of 47.5% acetonitrile, 5% water, and 5% formic acid, and finally 50 μL of acetonitrile/formic acid solution once again. The supernatants

from each of these sequential incubations were pooled, and sample volumes were reduced to ~15 μL using a speed-vac concentrator.

Samples were analyzed by reversed-phase high-performance liquid chromatography (HPLC) using a Thermo EASY-nLC 1200 HPLC equipped with a self-packed Aeris 1.7 μm C18 analytical column (0.075 mm by 14 cm, Phenomenex). Peptides were eluted using standard reverse-phase gradients and analyzed using a Thermo Q Exactive HF-X Hybrid Quadrupole-Orbitrap mass spectrometer (nanospray configuration). The resulting fragmentation spectra were correlated against the known peptide database using Scaffold (Proteome Software Inc.). Analyses in Supplemental Table S3 were performed using Scaffold.

Small-RNA blots

Total RNA was isolated from log-phase cultures (OD_{600} 0.8-1.0) of *N. castellii* using the hot-phenol method. 20 μg of total RNA was loaded per lane of a denaturing 15% polyacrylamide gel. After blotting, carbodiimide was used to crosslink small RNAs to the membrane (16). DNA probes (*N. castellii* palindrome-derived siRNA, 5′-CTATCTTCATCGATTACCATCTA; *N. castellii* Y′ siRNA, 5′-TCATGGTTAAGTATGGACGTCAA; *N. castellii* U6 small nuclear RNA, 5′-TATGCAGGGGAACTGCTGAT; *H. sapiens* miR-21, 5′-TCAACATCAGTCTGATAAGCTA) were labeled at their 5′ termini, as was the LNA probe for *A. thaliana* miR156 (17). Strains used in Figure 3C were DPB220 and DPB622. Strains used in Supplemental Figure S2A were DPB211, DPB443, DPB442, DPB620, DPB601, DPB608. Periodate oxidation and beta-elimination was as described (18).

Small-RNA sequencing, RNA-seq, and analysis

Genome and small-RNA cluster assembly

The *N. castellii* genome used for analysis of small-RNA and RNA sequencing from wild-type (DPB220), *AGO1* slicing-impaired (DPB228), and Δ*xrn1* (DPB622) strains in Figure 3 was downloaded from YGOB. The sequences of the Y′-consensus ORF (1) as well as the chloramphenicol and firefly luciferase internal-standard mRNA sequences were added to the FASTA file. The *N. castellii* genome used for analysis of RNA sequencing from wild-type (DPB537) and Δ*xrn1* (DPB541) genetic-selection strains in Supplemental Figures S2C–E and S5C was the *N. castellii* selection genome used for genome-sequencing analysis supplemented with the Y′-consensus ORF as well as the chloramphenicol and luciferase internal-standard mRNA sequences. The *N. castellii* genome used for RNA sequencing analysis with strains DPB537 and DPB541 in Supplemental Figure S5D was downloaded from Ensembl (ASM23734v1). STAR (v.2.4) was used to generate indexed genomes.

The coordinates of annotated small-RNA clusters (1) were converted from scaffolds of the unfinished *N. castellii* genome to the genomic coordinates of the finished *N. castellii* genome using the liftOver command line tool from UCSC (https://genome.ucsc.edu/). The sequence of one annotated small-RNA cluster did not match the finished genome, and thus this cluster was omitted. For some small-RNA clusters, the former scaffold coordinates overlapped in the finished genome; these were combined to generate larger clusters in the updated set of small-RNA clusters. In instances in which a region of a previously annotated small-RNA cluster was partially deleted in the new genome, the remainder was retained as a smaller cluster. In cases in which the scaffold sequence of a small-RNA cluster mapped to multiple sequences in the finished genome, only one set of coordinates for the cluster was retained. Several new clusters

were also identified as genomic regions for which 22–23-nt reads mapped but were not previously annotated as small-RNA clusters and did not overlap known rRNA or tRNA loci. The updated table of the genomic coordinates of the small-RNA clusters is provided (Supplemental Table S4).

Small-RNA read processing and expression analysis

The first four nucleotides of each sequencing read were removed using fastx_trimmer from the FASTX-toolkit (http://hannonlab.cshl.edu/fastx_toolkit/index.html). The 3′ adapter was then trimmed using cutadapt (v1.8) (19) with parameters '-a NNNNTCGTATGCCGTCTTCTGCTTG -m 9'. High-quality reads were then selected using fastq_quality_filter from the FASTX-toolkit with parameters '-v -q 30 -p 100 -Q 64 -z'. 9–26-nt, and 10–12-nt or 22–23-nt high-quality reads were selected using cutadapt. Reads were aligned to their appropriate *N. castellii* genomes using STAR (v.2.4) allowing no mismatches or gaps with the parameters '--runThreadN 30 --alignIntronMax 1 --alignIntronMin 2 --scoreDelOpen -10000 --scoreInsOpen -10000 --outFilterMismatchNmax 0 --seedSplitMin 9 --outFilterType BySJout --outFilterMultimapNmax 1000 --outSAMtype BAM SortedByCoordinate --readFilesCommand zcat --outFilterIntronMotifs RemoveNoncanonicalUnannotated --alignEndsType EndToEnd'. To remove rRNA and tRNA reads, featureCounts (20) was used to fractionally count the number of times each read mapped to annotated rRNA loci and their intergenic regions (Ncas_Chr3: 572204–588441) and to annotated tRNA loci in the *N. castellii* genome using the parameters '-s 1 -f -O -M --fraction --donotsort'. Additionally, featureCounts was used to produce a SAM file of these rRNA and tRNA reads which were then removed from the original files of 9–26-nt, 10–12-nt or 22–23-nt high quality reads using custom Python

scripts. The high-quality reads that were depleted of rRNA and tRNA reads were then aligned to the appropriate *N. castellii* genomes using STAR (v.2.4) with the same parameters as before. To fractionally count the number of times each read mapped to specific features in the *N. castellii* genome, featureCounts (20) was utilized using the parameters '-s 1 -f -O -M --fraction --donotsort'. For the small-RNA sequencing analysis with wild-type (DPB220), *AGO1* slicing-impaired (DPB228), and Δ*xrn1* (DPB622) strains, this fractional counting was performed using either a GTF file comprised of the entire genome divided into genic or intergenic regions or a GTF file of the previously annotated small-RNA clusters, each of which included the Y′-consensus ORF. For the small-RNA sequencing analysis with wild-type (DPB537) and Δ*xrn1* (DPB541) genetic-selection strains, this fractional counting was performed using a GTF file of the previously annotated small-RNA clusters, including the Y′-consensus ORF as well as the synthetic hairpins used in the genetic selection. Fractional counts were converted to reads per million for each locus and then normalized based on the internal small-RNA standard reads in each library. In Figure 3B, the 22–23-nt reads that mapped to the small-RNA clusters were extracted from the BAM file and categorized based on whether they mapped exclusively to palindromic or non-palindromic loci or if they mapped to both types of loci.

Analysis of passenger-strand stability

Analysis started with 22–23-nt and 10–12-nt reads that mapped to the small-RNA clusters. Custom Python scripts identified 22–23-nt reads that could pair perfectly with each other with the 2-nt 3′ overhangs characteristic of siRNA duplexes, as well as 10- or 11-nt reads that perfectly matched 5′ ends of these species and 12-nt reads that perfectly matched the 3′ ends of these species. Ambiguous pairs for which a 22–23-mer had multiple 22–23-mers that could

potentially pair to it but with different 2-nt 3′ overhangs were excluded. In instances for which a 10–12-mer matched more than one 22–23-mer, its number of reads was divided by the number of matching 22–23-mers and assigned equally to these 22–23-mers. Each guide-passenger pair displayed in Figure 5 passed the following cutoffs in the wild-type strain, the Δ*xrn1* strain, as well as the *AGO1* slicing-impaired strain: the guide strand had ≥100 reads; the full-length passenger strand had ≥1 read; and at least one of the two passenger-strand cleavage fragments had ≥1 read. Additionally, the ratio of full-length passenger-strand reads to guide-strand reads was greater in the *AGO1* slicing-impaired strain than in the WT strain, and the ratio of cleaved passenger-strand reads to guide-strand reads was greater in the WT strain than in the *AGO1* slicing-impaired strain.

RNA-seq read processing and expression analysis

RNA-seq reads were aligned to the genome using STAR (v.2.4) with the parameters '--outFilterType BySJout --outFilterMultimapNmax 1000 --outFilterMismatchNoverReadLmax 0.04 --alignIntronMax 1100 --outSAMtype BAM SortedByCoordinate --readFilesCommand zcat --outFilterIntronMotifs RemoveNoncanonicalUnannotated'. As with the small-RNA sequencing, featureCounts were used to fractionally count the number of times each read mapped to the genome using the parameters '-s 2 -f -O -M --fraction --donotsort'. This fractional counting was performed using a GTF file of the previously annotated small-RNA clusters, which included the Y′-consensus ORF. The fractional counts were converted to transcripts per million and normalized in the same manner as the small-RNA sequencing libraries. Browser tracks for visualizing RNA-seq results shown in Supplemental Figures S2B and S5C were made using IGV

(v.2.4.10) (21,22). The metagene profile shown in Supplemental Figure S5D was generated using ngs.plot.r (v.2.61) (23) using parameters '-R genebody -SE 0 -L 100'.

RNAs for in vitro assays

The guide RNA (5′-UAAAGUGCUUAUAGUGCAGGUAG), the passenger-strand RNA (5′-ACCUGCACUAUAAGCACUUUAAG), the passenger-strand RNA for 3′ labeling (5′-ACCUGCACUAUAAGCACUUUAA), and the slicing target (5′-GGGAGAAACAAAAAUACCUACCUGCACUAUAAGCACUUUACCAUCUCAAACUUACUCAGA) were synthesized, purified, and labeled as described (24) with the following adaptations. Capping and cap-labeling were performed with the Vaccinia Capping System (NEB). To generate unlabeled capped target, a large-scale capping reaction (60 μL reaction containing 60 μg target RNA) was performed and capped RNA was gel purified on a long, denaturing 12% polyacrylamide gel that resolved the capped and uncapped species.

Ago1p and Xrn1p protein purification

50 mL SR – Ura cultures (S – Ura media + 2% raffinose) were inoculated with cells from SD – Ura plates of *S. cerevisiae* strains DPB598 (which expressed WT *N. castellii* Ago1p in a Δ*xrn1* background), DPB1100 (which expressed WT *N. castellii* Xrn1p in a Δ*xrn1* background), DPB1101 (which expressed mutant *N. castellii* Xrn1p, containing D206N and D208N substitutions at the active site in a Δ*xrn1* background). 1000 mL liquid yeast extract, peptone (YEP) + 2% raffinose cultures were inoculated with cells from the 50 mL SR – Ura cultures at OD_{600} ~0.1. After growth at 30°C to OD_{600} 1.2, 100 mL of 20% galactose was added, and the cultures were incubated with shaking at 30°C for 24 h. Cultures were harvested by

centrifugation at 2000 x *g* for 5 min. The cells were washed once with 40 mL 0.1 mM 4-(2-aminoethyl)benzenesulfonyl fluoride hydrochloride (AEBSF, Sigma-Aldrich A8456), transferred into conical 50 mL tubes and centrifuged at 1700 x *g* for 5 min. The cells were washed once in 300 mM glutamate/sorbitol buffer (50 mM Hepes pH 7.6, 1 mM EGTA, 1 mM EDTA, 5 mM magnesium acetate, 10% glycerol, 300 mM potassium glutamate, 800 mM sorbitol, 1 mM DTT added fresh) and pelleted by centrifugation at 1500–1700 x *g* for 5–7 min, and then 1/3 cell volume of glutamate buffer with protease inhibitor (50 mM Hepes pH 7.6, 1 mM EGTA, 1 mM EDTA, 5 mM magnesium acetate, 10% glycerol, 300 mM potassium glutamate, 1 mM DTT added fresh, 1 cOmplete, Mini, EDTA-free Protease Inhibitor Cocktail (Roche 11836170001) tab per 10 mL buffer) was added. The cell slurry was dripped slowly into liquid nitrogen to form frozen beads of cells, which were stored at –80°C. The cells were lysed in a SPEX SamplePrep 6870 Freezer/Mill (10 cycles of 2 min grinding and 2 min cool down). After cell lysate was thawed on ice for 60 min, glutamate buffer with protease inhibitor was added (~5 mL for 5 g of cell powder) and lysate was centrifuged at 30,000 x *g* for 90 min at 4°C. The supernatant was collected and centrifuged at 30,000 x *g* for 30 min and then additional glutamate buffer with protease inhibitor was added (another 5 mL for 5 g of cell powder). The supernatant was added to ANTI-FLAG M2 Affinity Gel (Millipore A2220; adding 2 mL gel slurry for 5 g of cell powder) and incubated with rotation for 2.5 h at 4°C. The affinity agarose was washed twice with 1M glutamate/NP40 buffer (50 mM Hepes pH 7.6, 1 mM EGTA, 1 mM EDTA, 5 mM magnesium acetate, 10% glycerol, 1 M potassium glutamate, 0.01% NP-40, 5 mM DTT added fresh) and twice with 300 mM glutamate/NP40 buffer (50 mM Hepes pH 7.6, 1 mM EGTA, 1 mM EDTA, 5 mM magnesium acetate, 10% glycerol, 300 mM potassium glutamate, 0.01% NP-40, 5 mM DTT added fresh). Proteins were eluted by incubation of the affinity agarose with 300

mM glutamate buffer containing TEV protease (50 mM Hepes pH 7.6, 1 mM EGTA, 1 mM EDTA, 5 mM magnesium acetate, 10% glycerol, 300 mM potassium glutamate, 0.01% NP-40, 5 mM DTT added fresh, 25 μL TEV protease (Sigma-Aldrich T4455) per 1 mL buffer) for 15 h at 4°C. For the Ago1p purification, the TEV protease was removed using Ni-NTA Agarose (QIAgen, 30210) according to manufacturer's protocol. Concentrations of the purified proteins were determined by Bradford Assay using the Bio-Rad Protein Assay Dye Reagent Concentrate (Bio-Rad 5000006) and calculating the ratio of the samples' absorbance at 595 nm and 472 nm. Protein aliquots were flash frozen in liquid nitrogen and stored at –80°C.

RISC purification

Lysate of *S. cerevisiae* strain DPB598, prepared as described above, was incubated with an siRNA duplex containing 5′-radiolabeled miR-20a and the corresponding passenger strand (24) (final concentration, 0.25 μM) for 7 h at 30°C with shaking (600 RPM). Subsequent purification of miR-20a-loaded Ago1p-RISC was based on affinity to the miR-20a seed (25). Streptavidin agarose beads (Sigma-Aldrich S1638) were resuspended in storage buffer and 1.5 mL of slurry was washed three times with 300 mM glutamate/NP40 buffer and bound to 5 nmol capture 2′-*O*-methyl RNA oligonucleotide (5′-CUCACCUUCUACACCACC**GCACUUUA**UCCUUACACAC-3′-biotin; bold, match to the miR-20a extended seed region) in 1.5 mL of water by incubating for 90 min at 30°C. The agarose beads were then washed three times with 300 mM glutamate/NP40 buffer, incubated with lysate for 10 h at 4°C, and then washed three times with 1M glutamate/NP40 buffer and three times with 300 mM glutamate/NP40 buffer. Ago1p-RISC was then eluted by adding 10 nmol competitor DNA oligonucleotide (5′-

AAGGATAAAGTGCGGTGGTGTAGAAGGTGAG-3′) in 1 mL 300 mM glutamate/NP40 buffer to the agarose beads. After incubating for 2 h at 4°C, eluate was collected, and Ago1p-RISC was bound to and eluted from ANTI-FLAG M2 Affinity Gel as described above. Ago1p-RISC concentration was determined based on the fraction of the input radioactivity that remained associated with purified Ago1p-RISC. Protein aliquots were flash frozen in liquid nitrogen and stored at –80°C.

Supplemental Figure S1

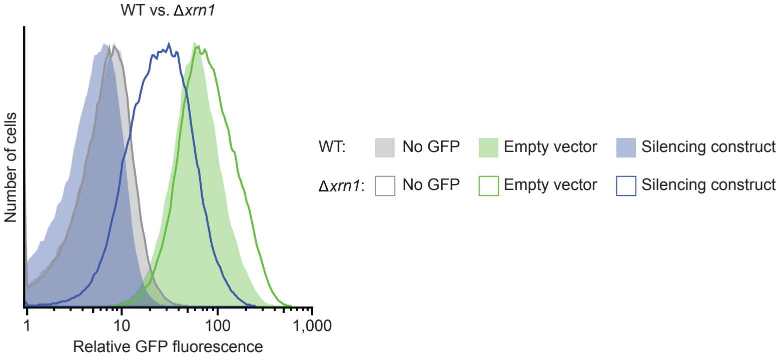

Supplemental Figure S2

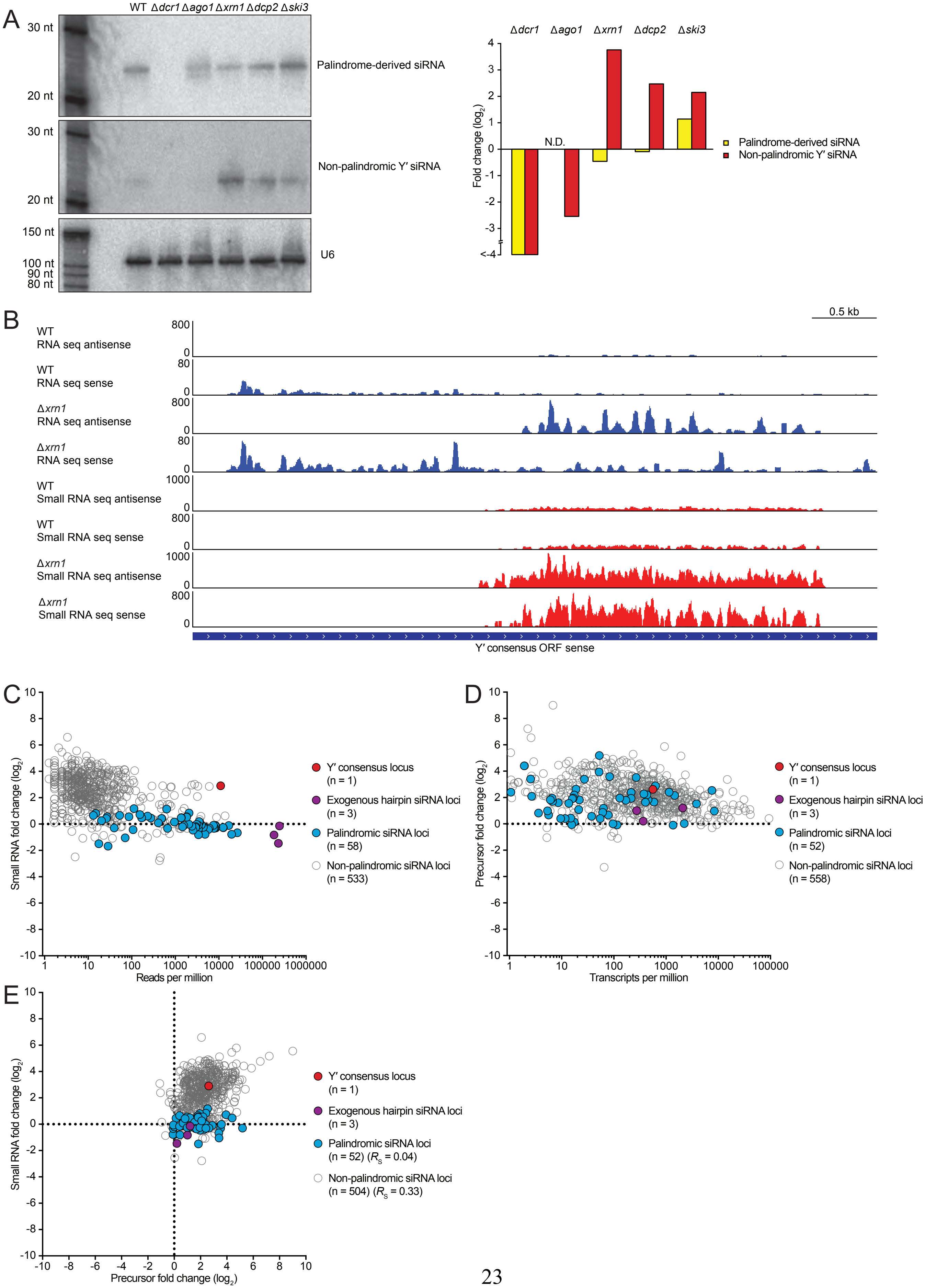

Supplemental Figure S3

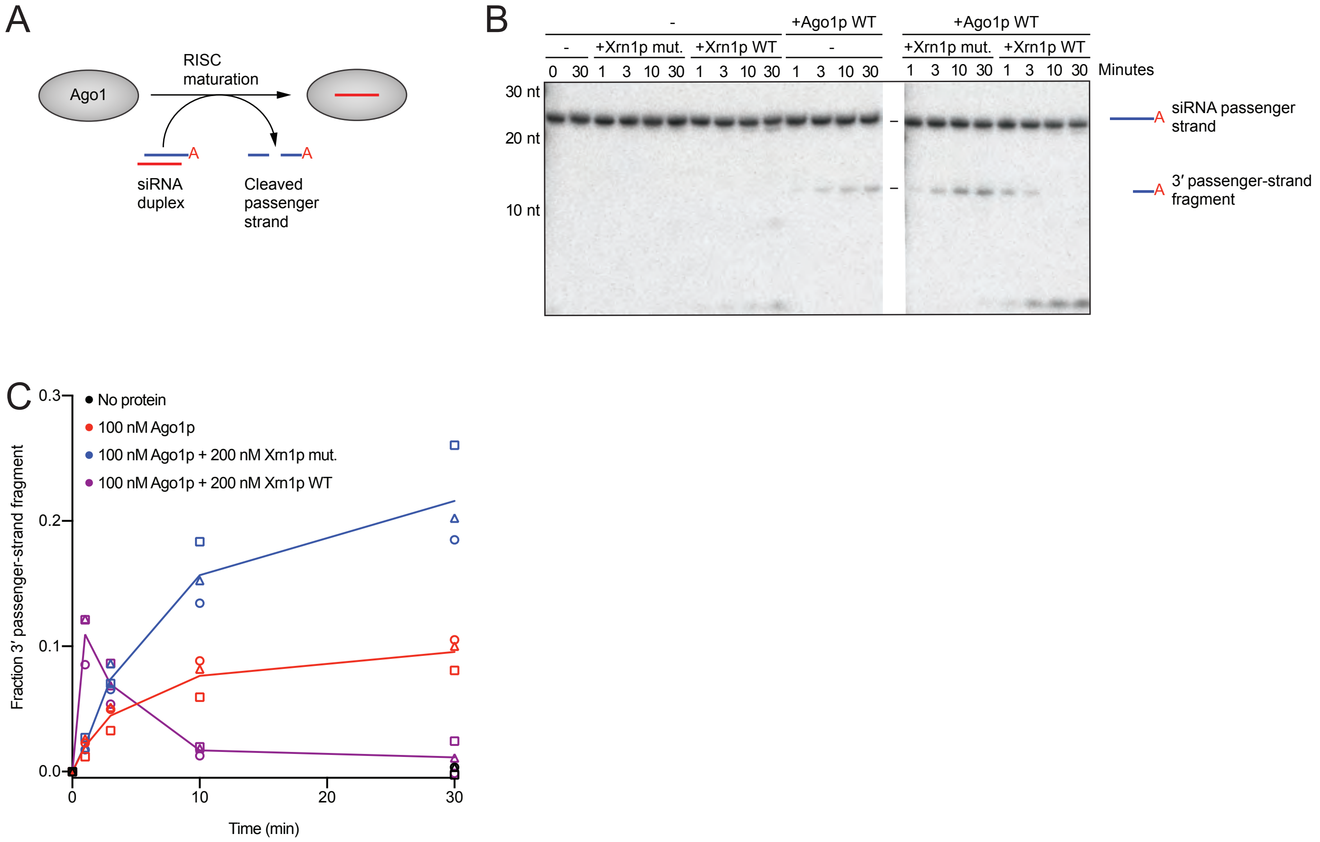

Supplemental Figure S4

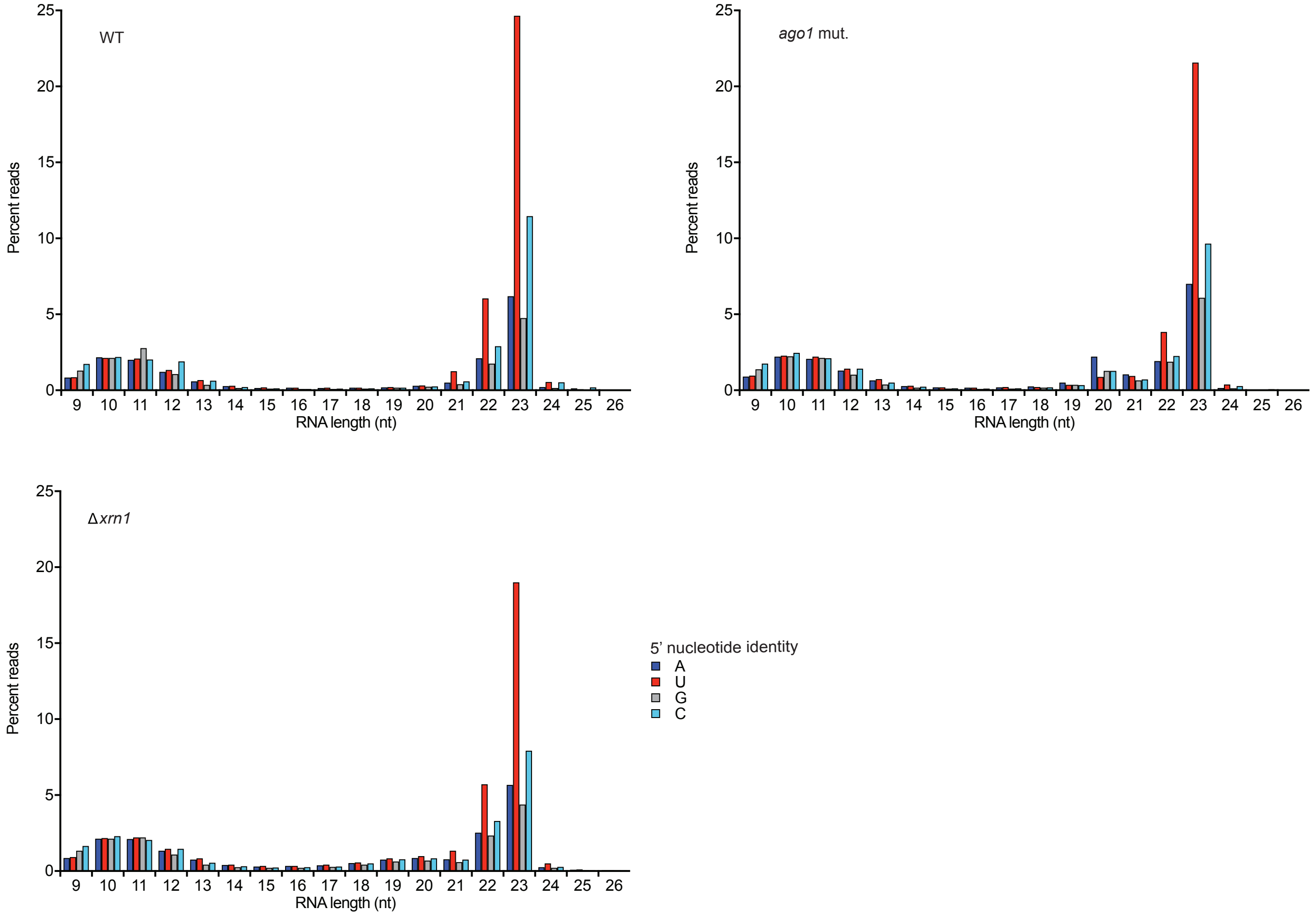

Supplemental Figure S5

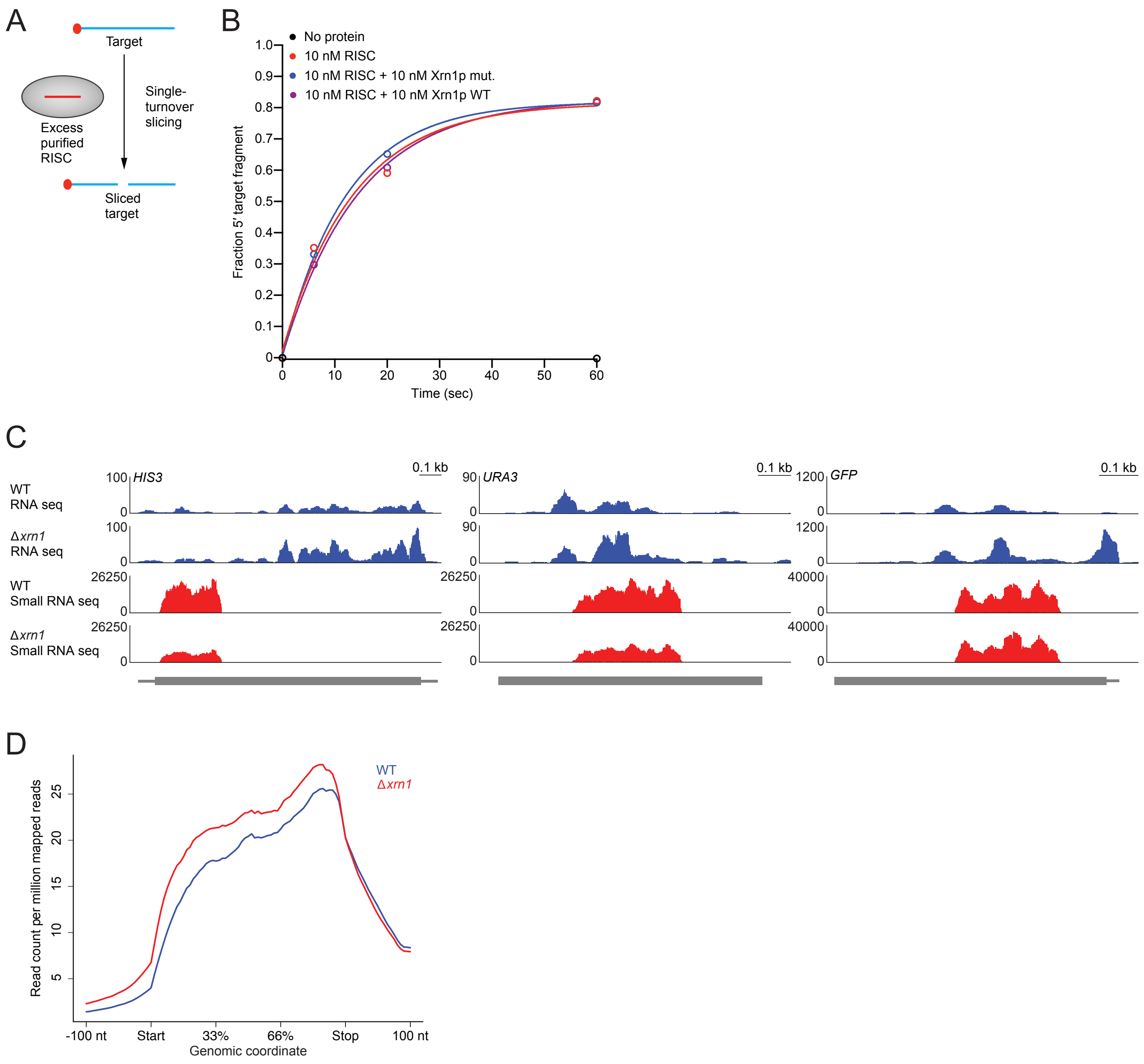

Supplemental Figure S6

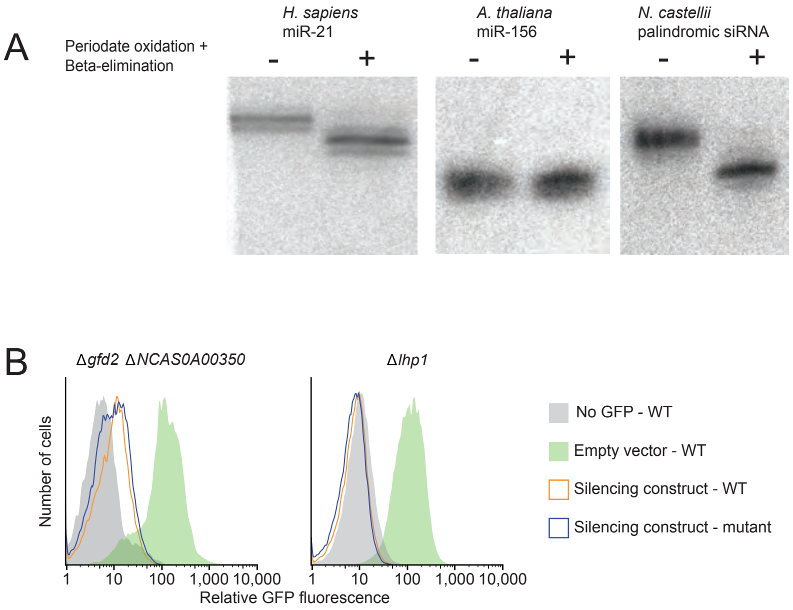

SUPPLEMENTAL FIGURE AND TABLE LEGENDS

Supplemental Figure S1. A large effect on *GFP* silencing observed upon disruption of *XRN1* in strains containing the silencing construct. Shown are overlaid histograms from **Figure 1E** of GFP fluorescence measured by flow cytometry of the WT (filled, shaded) and Δ*xrn1* (outline) haploid strains that either lacked the *GFP* gene (gray), contained *GFP* but had no silencing construct (green), or contained both *GFP* and a *GFP*-silencing construct (blue).

Supplemental Figure S2. The effects of Xrn1p and other proteins on the abundance of small RNAs and their precursors in *N. castellii*. **A)** Effects of RNAi proteins and several RNA-decay factors on the expression levels of both a palindrome-derived siRNA and a non-palindrome-derived siRNA. Shown are the results of successively probing an RNA blot, as in **Figure 3C** (N.D., no significant difference). In contrast to most siRNAs in the Δ*ago1* strain (1), the palindrome-derived siRNA probed in this experiment is relatively stable. **B)** Effects of Xrn1p on the Y′ consensus ORF and on siRNAs targeting the Y′ ORFs. Profiles for strand-specific RNA-seq (blue) and small-RNA sequencing (red) are shown for the Y′ consensus ORF in WT and Δ*xrn1* strains. The polarity of the sense strand of the Y′ consensus ORF is schematized below the profiles, with a blue line with white arrow heads pointing from 5′ to 3′. Track heights were normalized based on reads that mapped to the internal standards, with the RNA-seq reads for the WT strain normalized with respect to reads for the Δ*xrn1* strain and the small-RNA sequencing reads for the Δ*xrn1* strain normalized with respect to those of the WT strain. **C)** Effect of Xrn1p on the abundance of small RNAs originating from siRNA-producing loci of the *N. castellii* genetic-selection strain. Otherwise, as in **Figure 3A**. **D)** Effect of Xrn1p on the abundance of small-RNA precursor transcripts in the *N. castellii* genetic-selection strain. Otherwise, as in

Figure 3D. **E)** Relationship between the changes in small RNAs and the changes in siRNA precursor transcripts observed after deleting *XRN1* in the *N. castellii* genetic selection strains. Otherwise, as in **Figure 3E**.

Supplemental Figure S3. Impact of Xrn1p on stability of the 3′ passenger-strand cleavage fragment. **A)** Experimental scheme of the assay of duplex loading and passenger-strand cleavage. The red A in the duplex indicates a radiolabeled cordycepin at the 3′-end of the passenger strand. The initial concentration of siRNA duplex was 10 nM. **B)** Effect of Xrn1p on passenger-strand cleavage and degradation in the assay schematized in **A**. Shown is a representative denaturing gel resolving 3′-labeled passenger strand from its cleavage product after incubation with or without purified Ago1p (100 nM) and with or without Xrn1p (WT or mut.) (200 nM) for the indicated amount of time. **C)** Quantification of the 3′ passenger-strand fragment. Otherwise, as in **Figure 4C**.

Supplemental Figure S4. The length distribution of small-RNA reads from WT, slicing-impaired *ago1* mutant, and Δ*xrn1 N. castellii* strains. At each length from 9–26 nt, the number of genome-matching sequencing reads with the indicated 5′ nucleotide is plotted. Reads mapping to either rRNA or tRNA loci were excluded.

Supplemental Figure S5. Effect of Xrn1p on single-turnover slicing and on accumulation of 3′ fragments of RNAi targets in vivo. **A)** Experimental scheme of single-turnover slicing of a cap-labeled target. The initial target concentration is substantially below that of RISC, otherwise as in **Figure 6A**. **B)** Quantification of the 5′ product of slicing under single-turnover conditions, as

schematized in **A**. Slicing was carried out in single-turnover conditions, incubating cap-labeled target (1 nM) with purified RISC–miR-20a (10 nM), with or without Xrn1p (WT or mut.) (10 nM) for the indicated amount of time. Results are shown for one experiment with either no protein (black), RISC–miR-20a only (red), RISC–miR-20a with Xrn1p mut. (blue), and RISC–miR-20a with Xrn1p WT (purple). Fraction cleaved was calculated by dividing the signal of product / (product + substrate). Lines represent the best fit to the equation for one-phase association. **C)** Effects of Xrn1p on mRNAs targeted by RNAi in the genetic selection and on siRNAs targeting these mRNAs. Strand-specific RNA-seq (blue) and small-RNA sequencing (red) profiles are shown for mRNAs targeted by RNAi (*GFP*, *HIS3, URA3*) in WT and Δ*xrn1* genetic-selection strains. The mRNAs are schematized below the profiles, with thick gray lines indicating coding sequence and thin gray lines indicating untranslated regions. Track heights were normalized as in **Supplemental Figure S2B**. **D)** The effect of Xrn1p on the mRNAs of non-RNAi targets. Shown are metaplots of RNA-seq reads mapping to mRNAs that were not targets of RNAi in WT (blue) and Δ*xrn1* (red) genetic-selection strains. The start and stop codons are indicated, as are positions 100 nucleotides (nt) upstream and downstream of the ORF. Reads displayed are normalized to library size.

Supplemental Figure S6. Assessment of siRNA 2′-O-methylation and the effect of disrupting RNAi cofactor orthologs in *N. castelii*. **A)** Examination of 2′-O-methylation of the 3′-terminal nucleotide of *N. castellii* siRNAs. Total RNA from a human cell line (HeLa), *Arabidopsis thaliana* leaves, and *N. castellii* was subjected to periodate oxidation and beta-elimination. Samples with and without treatment were then analyzed on RNA blots. The human sample was probed for miR-21, which is not 2′-O-methylated and is therefore susceptible to oxidation and

elimination, the *A. thaliana* was probed for miR-156, which is 2′-O-methylated and therefore protected from oxidation and elimination, and the *N. castellii* sample was probed for a palindromic siRNA (as in **Figure 3C**). **B)** Effect of disrupting the orthologs of genes reported to enhance RNAi in other species. Shown are histograms of GFP fluorescence measured by flow cytometry in Δ*gfd2* Δ*NCAS0A00350* and Δ*lhp1 N. castellii* strains with the indicated GFP-silencing constructs. All strains were induced.

Supplemental Table S1. Protein-coding or tRNA mutations identified in each mutant strain sequenced. For each mutation, the gene, the mutant sequence, the genomic coordinate, and the reference sequence are listed.

Supplemental Table S2. Genes mutated in the mutant strains that were sequenced. For each gene, the number of sequenced strains that had mutations in the gene is indicated, as well as the total number of mutations and the number of unique and nonsense mutations.

Supplemental Table S3. Ago1p immunoprecipitation and mass spectrometry results for two separate experiments. The tables were each sorted by enrichment of total spectra in the pull-down condition over the control condition.

Supplemental Table S4. Updated genomic coordinates of siRNA-producing transcripts.

Supplemental Table S5. Strains used and generated in this study.

Supplemental Table S6. Plasmids generated in this study.

SUPPLEMENTAL REFERENCES

1. Drinnenberg, I.A., Weinberg, D.E., Xie, K.T., Mower, J.P., Wolfe, K.H., Fink, G.R. and Bartel, D.P. (2009) RNAi in budding yeast. *Science*, **326**, 544-550.
2. Chin, B.L., Ryan, O., Lewitter, F., Boone, C. and Fink, G.R. (2012) Genetic variation in Saccharomyces cerevisiae: circuit diversification in a signal transduction network. *Genetics*, **192**, 1523-1532.
3. Sikorski, R.S. and Hieter, P. (1989) A system of shuttle vectors and yeast host strains designed for efficient manipulation of DNA in Saccharomyces cerevisiae. *Genetics*, **122**, 19-27.
4. Szewczyk, E., Nayak, T., Oakley, C.E., Edgerton, H., Xiong, Y., Taheri-Talesh, N., Osmani, S.A. and Oakley, B.R. (2006) Fusion PCR and gene targeting in Aspergillus nidulans. *Nat Protoc*, **1**, 3111-3120.
5. Brachmann, C.B., Davies, A., Cost, G.J., Caputo, E., Li, J., Hieter, P. and Boeke, J.D. (1998) Designer deletion strains derived from Saccharomyces cerevisiae S288C: a useful set of strains and plasmids for PCR-mediated gene disruption and other applications. *Yeast*, **14**, 115-132.
6. Guldener, U., Heck, S., Fielder, T., Beinhauer, J. and Hegemann, J.H. (1996) A new efficient gene disruption cassette for repeated use in budding yeast. *Nucleic Acids Res*, **24**, 2519-2524.
7. Krawchuk, M.D. and Wahls, W.P. (1999) High-efficiency gene targeting in Schizosaccharomyces pombe using a modular, PCR-based approach with long tracts of flanking homology. *Yeast*, **15**, 1419-1427.
8. Goldstein, A.L. and McCusker, J.H. (1999) Three new dominant drug resistance cassettes for gene disruption in Saccharomyces cerevisiae. *Yeast*, **15**, 1541-1553.
9. Longtine, M.S., McKenzie, A., 3rd, Demarini, D.J., Shah, N.G., Wach, A., Brachat, A., Philippsen, P. and Pringle, J.R. (1998) Additional modules for versatile and economical PCR-based gene deletion and modification in Saccharomyces cerevisiae. *Yeast*, **14**, 953-961.
10. Hailey, D.W., Davis, T.N. and Muller, E.G. (2002) Fluorescence resonance energy transfer using color variants of green fluorescent protein. *Methods Enzymol*, **351**, 34-49.
11. Randell, J.C., Bowers, J.L., Rodriguez, H.K. and Bell, S.P. (2006) Sequential ATP hydrolysis by Cdc6 and ORC directs loading of the Mcm2-7 helicase. *Mol Cell*, **21**, 29-39.
12. Vyas, V.K., Barrasa, M.I. and Fink, G.R. (2015) A Candida albicans CRISPR system permits genetic engineering of essential genes and gene families. *Sci Adv*, **1**, e1500248.
13. Vyas, V.K., Bushkin, G.G., Bernstein, D.A., Getz, M.A., Sewastianik, M., Barrasa, M.I., Bartel, D.P. and Fink, G.R. (2018) New CRISPR Mutagenesis Strategies Reveal Variation in Repair Mechanisms among Fungi. *mSphere*, **3**.
14. Dobin, A., Davis, C.A., Schlesinger, F., Drenkow, J., Zaleski, C., Jha, S., Batut, P., Chaisson, M. and Gingeras, T.R. (2013) STAR: ultrafast universal RNA-seq aligner. *Bioinformatics*, **29**, 15-21.
15. Li, H., Handsaker, B., Wysoker, A., Fennell, T., Ruan, J., Homer, N., Marth, G., Abecasis, G., Durbin, R. and Genome Project Data Processing, S. (2009) The Sequence Alignment/Map format and SAMtools. *Bioinformatics*, **25**, 2078-2079.

16. Pall, G.S., Codony-Servat, C., Byrne, J., Ritchie, L. and Hamilton, A. (2007) Carbodiimide-mediated cross-linking of RNA to nylon membranes improves the detection of siRNA, miRNA and piRNA by northern blot. *Nucleic Acids Res*, **35**, e60.
17. Nodine, M.D. and Bartel, D.P. (2010) MicroRNAs prevent precocious gene expression and enable pattern formation during plant embryogenesis. *Genes & Development*, **24**, 2678-2692.
18. Yu, B. and Chen, X. (2010) Analysis of miRNA Modifications. *Methods Mol Biol*, **592**, 137-148.
19. Martin, M. (2011) Cutadapt removes adapter sequences from high-throughput sequencing reads. *EMBnet.journal*, ***17*****(1)**, 10-12.
20. Liao, Y., Smyth, G.K. and Shi, W. (2014) featureCounts: an efficient general purpose program for assigning sequence reads to genomic features. *Bioinformatics*, **30**, 923-930.
21. Robinson, J.T., Thorvaldsdóttir, H., Winckler, W., Guttman, M., Lander, E.S., Getz, G. and Mesirov, J.P. (2011) Integrative genomics viewer. *Nat Biotechnol*, **29**, 24-26.
22. Thorvaldsdóttir, H., Robinson, J.T. and Mesirov, J.P. (2013) Integrative Genomics Viewer (IGV): high-performance genomics data visualization and exploration. *Brief Bioinform*, **14**, 178-192.
23. Shen, L., Shao, N., Liu, X. and Nestler, E. (2014) ngs.plot: Quick mining and visualization of next-generation sequencing data by integrating genomic databases. *BMC Genomics*, **15**, 284.
24. Nakanishi, K., Weinberg, D.E., Bartel, D.P. and Patel, D.J. (2012) Structure of yeast Argonaute with guide RNA. *Nature*, **486**, 368-374.
25. Flores-Jasso, C.F., Salomon, W.E. and Zamore, P.D. (2013) Rapid and specific purification of Argonaute-small RNA complexes from crude cell lysates. *RNA*, **19**, 271-279.
